# *In Vivo* Performance and Properties of Tamoxifen Metabolites for CreERT2 Control

**DOI:** 10.1371/journal.pone.0152989

**Published:** 2016-04-14

**Authors:** Anastasia Felker, Susan Nieuwenhuize, Aymeric Dolbois, Kristyna Blazkova, Christopher Hess, Larry W. L. Low, Sibylle Burger, Natasha Samson, Tom J. Carney, Petr Bartunek, Cristina Nevado, Christian Mosimann

**Affiliations:** 1 Institute of Molecular Life Sciences, University of Zürich, Zürich, Switzerland; 2 Department of Chemistry, University of Zürich, Zürich, Switzerland; 3 CZ-OPENSCREEN, Institute of Molecular Genetics of the ASCR, v.v.i., Prague, Czech Republic; 4 Institute of Molecular and Cell Biology (IMCB), A*STAR, Biopolis Drive, Singapore, Singapore; 5 Lee Kong Chian School of Medicine, Nanyang Technological University, 50 Nanyang Avenue, Singapore, Singapore; National University of Singapore, SINGAPORE

## Abstract

Mutant Estrogen Receptor (ERT2) ligand-binding domain fusions with Cre recombinase are a key tool for spatio-temporally controlled genetic recombination with the Cre/*lox* system. CreERT2 is efficiently activated in a concentration-dependent manner by the Tamoxifen metabolite *trans*-4-OH-Tamoxifen (*trans*-4-OHT). Reproducible and efficient Cre/*lox* experimentation is hindered by the gradual loss of CreERT2 induction potency upon prolonged storage of dissolved *trans*-4-OHT, which potentially results from gradual *trans*-to-*cis* isomerization or degradation. Here, we combined zebrafish CreERT2 recombination experiments and cell culture assays to document the gradual activity loss of *trans*-4-OHT and describe the alternative Tamoxifen metabolite Endoxifen as more stable alternative compound. Endoxifen retains potent activation upon prolonged storage (3 months), yet consistently induces half the ERT2 domain fusion activity compared to fresh *trans*-4-OHT. Using ^1^H-NMR analysis, we reveal that *trans*-4-OHT isomerization is undetectable upon prolonged storage in either DMSO or Ethanol, ruling out isomer transformation as cause for the gradual loss of *trans*-4-OHT activity. We further establish that both *trans*-4-OHT and Endoxifen are insensitive to light exposure under regular laboratory handling conditions. We attribute the gradual loss of *trans*-4-OHT potency to precipitation over time, and show that heating of aged *trans*-4-OHT aliquots reinstates their CreERT2 induction potential. Our data establish Endoxifen as potent and reproducible complementary compound to 4-OHT to control ERT2 domain fusion proteins *in vivo*, and provide a framework for efficient chemically controlled recombination experiments.

## Introduction

Temporal control of Cre recombinase for *lox* recombination genetics is commonly achieved by fusing Cre with the T2 mutant form of the Estrogen Receptor (ER) ligand-binding domain (CreERT2) that retains Cre in the cytoplasm until chemical induction triggers nuclear import [1]. This elegant mechanism is based on the ERT2 mutant’s insensitivity to its original natural ligand 17β-oestradiol (E2) and high affinity for synthetic estrogen mimics, including metabolites of the pro-drug Tamoxifen (Tam) [1,2] or the synthetic Cyclofen [3] and Faslodex (ICI 182,780) [4].

*In vivo*, Cytochrome p450 enzymes including the isoforms CYP2D6 and CYP3A4 convert Tam into the predominant active metabolites 4-Hydroxy-Tamoxifen (4-OHT) and N-desmethyl-4-hydroxytamoxifen (Endoxifen) [5], and both metabolites have up to 100 times stronger affinity to the ER domain than Tam itself [6,7]. 4-OHT acts as ER antagonist and inhibits target gene transcription by ER. While the majority of studies on ER-dependent breast cancer and genetic CreERT2 experiments have focused on 4-OHT, Endoxifen also potently interacts with the ER ligand-binding domain. Endoxifen results from enzymatic conversion of either Tam or 4-OHT and accumulates at higher titers than 4-OHT in the blood of Tam-treated patients, suggesting either faster metabolic kinetics or increased stability *in vivo* [8–10]. Underlining its potency, Endoxifen has been successfully applied in ER-based tumor treatment of patients with deficient CYP2D6 metabolism that precludes classic Tam treatment [11].

For recombination genetics in mice, intra-peritoneal injections or feeding of Tam into CreERT2-expressing transgenic mice are the routine protocols for recombination induction. The required metabolic conversion of Tam into its active metabolites introduces a lag time in *lox* recombination [2]. In contrast, similar to cell culture-based approaches, zebrafish CreERT2 experiments in embryos can be performed by directly adding 4-OHT to the embryo medium [12–14]. With potent CreERT2-expressing transgenes, such treatment rapidly induces detectable *lox* recombination [12], suggesting fast absorption into the embryos. Cyclofen has been shown to function akin to 4-OHT in zebrafish and is easily synthesized with the added capacity to generate laser-inducible, caged Cyclofen for single-cell *lox* recombination experiments [3].

Structurally, both 4-OHT and Endoxifen can exist as *cis* and *trans* isomers. *Trans*-4-OHT binds the ER ligand-binding domain with over 300-fold higher affinity than the *cis* isomer and is therefore the preferred active metabolite for ER interactions [7]. It is important to note that the *trans* isomer, in which the alkyl and the arylalkylamino side chains are in opposite sides of the double bound, corresponds to the “*Z*” isomer according to the Cahn-Ingold-Prelog rules.

Simple chemical handling and stability of the used compounds are critical parameters to reproducible CreERT2 experiments. We and others had previously noted that while undissolved *trans*-4-OHT powder remains stable in the dark at 4° C, working *trans*-4-OHT solutions of 10 mM in either DMSO or Ethanol drop in their potency to induce CreERT2-mediated *lox* recombination within weeks of storage at -20° C or -80° C [15]. The source of this instability remains unidentified. Isomerization of methanolic solutions of *trans*-4-OHT upon exposure to sunlight for at least 24 h has been reported [16]; consequently, accumulation of *cis*-4-OHT in the initially highly pure *trans*-4-OHT stocks (>98%) proposes a possible explanation for the loss of potency over time. Katzenellenbogen et al. observed up to 25% isomerization of *cis*-4-OHT to the *trans*-isomer in cell culture conditions at 37°C after 48 h while *trans*-4-OHT was also susceptible to the isomerization into the *cis*-isomer in a comparable extent (17%) [7]. In CDCl_3_ solutions, 4-OHT samples undergo a facile isomerization to a mixture of (*Z/E*)-4-hydroxytamoxifen, presumably due to the presence of traces of acid or by a bimolecular oxidative-reductive reaction that might result in a single bond rotation [17]. Work on chemically related, yet not identical, stilbene compounds proposed that *cis*-*trans* isomerization of Tam derivatives might be highly light-sensitive, and *trans*-4-OHT indeed rapidly isomerizes and hydrolyses upon UV laser exposure [3,18]. Conversely, *trans*-4-OHT working stocks for recombination experiments are commonly stored in the dark, and bench-top handling does not expose the solutions to intensive UV light for extended times.

These contradicting observations of the chemical properties of *trans*-4-OHT have dictated currently used laboratory protocols of *trans*-4-OHT handling. Reproducible experimentation mandates the generation of one-time working aliquots of *trans*-4-OHT in DMSO or Ethanol that need to be used within 4–6 weeks of their preparation, or alternatively the generation of newly dissolved *trans*-4-OHT directly from powder for each experiment [13,19]. Chemically, systematic experimental data and detailed elucidation of the extent to which these effects impact *trans*-4-OHT in genetic experiments is critically missing. In addition to more reproducible *trans*-4-OHT handling and storage, an alternative compound that i) remains stable in stock solution, ii) allows simple laboratory handling and availability, and iii) retains strong potency to induce CreERT2 recombination activity would be highly desirable.

Here, we combined *in vivo* and *in vitro* activity assays with chemical characterization towards improving experimental consistency of CreERT2 experiments in zebrafish. We compared the properties of *trans*-4-OHT versus Endoxifen under standard laboratory storage conditions over time for *trans-cis* isomerization and degradation, and tested CreERT2 recombination induction in developing zebrafish embryos. We find that Endoxifen efficiently triggers CreERT2 activity in zebrafish and translocates ERT2 domain reporters in cell culture at concentrations commonly used for *trans*-4-OHT, yet at consistently lower potency compared to *trans*-4-OHT. In contrast, while *trans*-4-OHT loses its induction potency within weeks of preparing stock solutions, Endoxifen remains stable for months under proper storage. ^1^H-NMR analysis revealed that commercially available active Endoxifen contains a 45:55 mixture of isomers and neither *trans*-4-OHT nor Endoxifen undergo significant isomerization upon storage, ruling out isomerization as cause for the gradual loss of *trans*-4-OHT potency. Our findings suggest that *trans*-4-OHT stocks in both DMSO and Ethanol gradually precipitate, *de facto* decreasing the active concentration. Simple heating or sonication of aged low-potency *trans*-4-OHT stocks partially reconstitutes their activity. In contrast, even after months of storage, Endoxifen efficiently induces CreERT2-mediated *lox* cassette recombination in developing zebrafish. Altogether, our work provides first experimental data on the stereoisomeric stability of stored dissolved Tam derivatives and establishes Endoxifen as slightly less potent but more consistent alternative for *trans*-4-OHT in standard recombination experiments and the control of ER-domain fusion proteins.

## Methods

### Zebrafish husbandry and genetics

Animal care and all experimental procedures were carried out in accordance with the European Communities Council Directive (86/609/EEC), according to which all embryo experiments performed before 120 hours post fertilization are not considered animal experimentation and do not require ethics approval. Adult zebrafish for breeding were kept and handled according to animal care regulation of the Kantonale Veterinäramt Zürich (TV4209). All zebrafish were raised, kept, and handled essentially as described [20]. Female individuals of the *ubi*:*Switch* line [13] were crossed with male transgenic zebrafish carrying *ubi*:*creERT2* (expressing *myl7*:*EGFP* as transgenic marker) [13] or *drl*:*creERT2* (expressing *α-crystallin*:*Venus* as transgenic marker) [21]. To assure same-staged embryos, adults were separated before mating by dividers. The eggs were collected, mixed to ensure a homologous diversity of clutches, and incubated in E3 medium at 28°C.

### Drug storage and administration

(Z)-4-Hydroxytamoxifen (H7904) and (E/Z)-Endoxifen Hydrochloride Hydrate (E8284) were obtained from Sigma Aldrich and stored at -20°C in the dark upon arrival and prior to dissolving. For each compound, 5 mg of the commercial powder were dissolved by continuously vortexing for ~15 minutes in 1) 1.29 ml and 1.22 ml of DMSO respectively, to reach a concentration of 10 mM or 2) 2.58 ml and 2.44 ml for 5 mM stocks. The solution was divided in 12 μl aliquots and transferred to 1.5 ml Eppendorf tubes. Both the aliquots and the remaining powder were stored in the dark at -20°C. At developmental stages of 50% epiboly or shield for *ubi*:*creERT2* x *ubi*:*Switch* and *drl*:*creERT2* x *ubi*:*Switch* crosses, respectively, the embryos were divided over 6-well plates, approximately 35 embryos per well. The single-use aliquots of the candidate compounds were thawed directly before administration to the embryos and 10 μl of the stocks were added to 10 ml E3 medium to obtain 10 μM induction medium. For the heating and sonication experiment, the single-use aliquots were heated at 65°C in a shaking thermoblock or sonicated in a sonication water bath for 10 min prior to addition to E3. For 0.1 μM inductions, the stock solutions were diluted 1:100 in DMSO and 10 μl of the diluted stock added to 10 ml of E3 medium. The controls received 10 μl of DMSO in 10 ml E3. The induction medium was vortexed thoroughly and 5 ml directly added to each well after removal of all E3 medium.

### Cell culture

U2OS cells (ATCC/LGC) cultured in standard DMEM (10% FBS, GlutaMAX) were stably transfected with pEGFP-ERT2, that was generated by subcloning of *ERT2* from *pCMV*:*CreERT* [2] to *pEGFP-C1* plasmid (Clontech). Clones with the highest EGFP fluorescence were selected using flow cytometry and used for the experimental work. Cells were transferred to starvation medium (phenol red-free DMEM, 4% charcoal-treated HyClone serum, GlutaMAX) 24–30 hours prior to the experiment. Cells were then transferred to a 384-well plate at 5000 cells/well and then treated with different concentrations of E2, 4-OHT and Endoxifen (DMSO solutions). After 20 hours at 37°C and 5% CO_2_ cells were stained using Hoechst 33342 for 20 minutes and images were taken from multiple fields per well using the high-content imaging system Operetta (Perkin Elmer).

### Imaging and analysis

The drug-treated embryos were analyzed at 4 dpf. Double-positive embryos (*myl7*:*EGFP*; *ubi*:*EGFP* for the *ubi*:*creERT2* crosses, *α-CY*:*Venus*; *ubi*:*EGFP* positive for *drl*:*creERT2* crosses) were separated and paralyzed with 0.016% Tricaine in E3. Images were taken with a Leica M205 fluorescent microscope and a Leica DFC450 C camera applying the same imaging settings to all embryos. The mCherry intensity was quantified with ImageJ, subtracting the fluorescent intensity of the background from the measured intensity of the whole image. Results (total area, mean, minimum value, maximum value, integrated density, fraction of area, raw integrated density) were exported to Excel. For every picture, the CTCF (corrected total cell fluorescence) was calculated by the formula ‘Integrated density whole–(area whole embryo * mean fluorescence background)’. This formula is loosely based on a method described for calculating cell-fluorescence [22] and applied using the following script:

run("Set Scale…", "distance = 7792.2003 known = 1 pixel = 1 unit = cm global");

//run("Channels Tool…");

Stack.setDisplayMode("grayscale");

Stack.setChannel(3);

//setTool("rectangle");

makeRectangle(4, 6, 2560, 1920);

run("Set Measurements…", "area mean min integrated area_fraction redirect = None decimal = 3");

run("Measure");

makeRectangle(2002, 1528, 558, 392);

run("Measure");

To control for imaging bias between different rounds of experiments (i.e. illumination power variations), relative CTCFs were calculated by setting all CTCFs of one round in relation to the highest CTCF obtained by *trans*-4-OHT treatments in the same experiment (assuming the highest CTCF represents the highest possible recombination result). Relative CTCFs for the heating/sonicating experiment were calculated in relation to the highest results for heated or sonicated compounds of the respective round of experiments.

Images of EGFP-ERT2 cells were analyzed using Columbus software (Perkin Elmer) using standard algorithms for cytoplasm and nuclei identification. Fluorescence intensity of EGFP was calculated for each of the regions. The ratio of mean nuclear EGFP fluorescence intensity to the mean fluorescence of the cytoplasm was calculated and curves were fitted using GraphPad Prism software.

### SPIM imaging

SPIM images were obtained with a Zeiss Lightsheet Z.1 microscope at ZMB, University of Zürich. Embryos were embedded in a rod of 1% low melting agarose in E3 with 0.016% Tricaine by using a syringe and plunger as described in the Zeiss manual for sample preparation for lightsheet imaging. Dual side imaging was performed and images from both illumination sources fused using the Zeiss Zen software. Maximum intensity projections were constructed by the Zeiss Zen software.

### Transverse vibratome sections

Embryos were fixed in 4% paraformaldehyde (PFA) overnight at 4°C. The fixed embryos were washed in PBS three times and embedded in 4% low-melt agarose in PBS/0.1% Tween-20. Sections of 100 μm thickness were performed with a Leica VT 1000S vibratome. Sections were mounted with DAPI-containing VectaShield (H-1200, Vector Laboratories) and imaged with a Zeiss LSM710 confocal microscope using a 40x oil objective.

### NMR analysis

*Trans*-4-OHT (1.55 mg) and Endoxifen (1.64 mg) were dissolved in 400 μl of DMSO-d_6_ or EtOD-d_6_ to reach a concentration of 10 mM. The solutions were transferred to an NMR tube covered with aluminum foil and stored at -20°C. The tubes were warmed up to room temperature prior to analysis and then put back at -20°C. Several aliquots of *trans*-4-OHT prepared according to the drug storage and administration paragraph were combined in one single 1.5 mL Eppendorf tube. Three aliquots (50 uL each) were transferred into three new Eppendorf tubes which were 1) untreated, 2) heated at 65°C for 10 min, or 3) sonicated for 15 min. DMSO-d6 was added (final volume 450 μL) and 1H-NMR of the samples was recorded. A fourth aliquot was sonicated in a glass vial for 15 min prior to dilution with DMSO-d6. ^1^H-NMR spectra were recorded on an AV2 400 MHz and AV2 500 MHz Bruker spectrometer. Chemical shifts are given in ppm. The spectra are calibrated to the residual ^1^H signals of the solvents.

### Statistical analysis

Statistical tests were performed with GraphPad Prism 5.03. To compare the means of the different concentrations of the two compounds or of activity of fresh and old stocks and the difference in heated *trans*-4-OHT potency in cell culture, two-way ANOVA was performed. To compare the activity of both compounds when fresh or *trans*-4-OHT activity after heating or sonication, we used a two-tailed, unpaired t-test. Statistical significance was determined by a p-value ≤ 0.05 (ns p>0.05, * p ≤ 0.05, ** ≤ 0.01, *** ≤ 0.001).

## Results

### Comparative activity analysis of Tamoxifen derivatives for ERT2 fusion protein modulation

**Fig 1:**
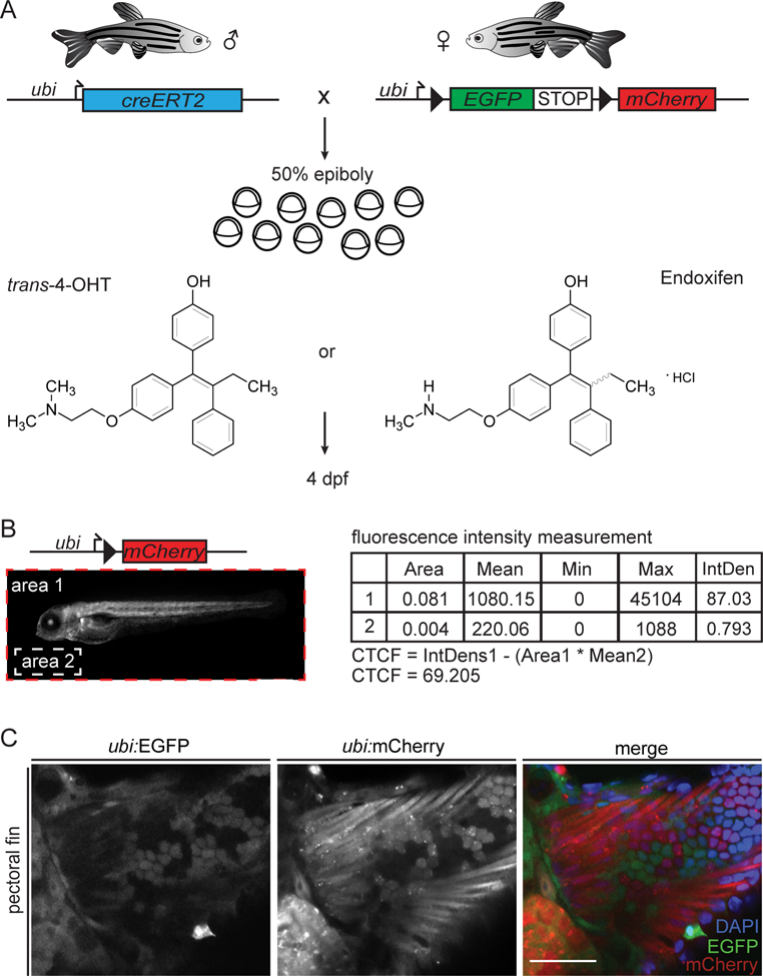
Experimental workflow to assess CreERT2 induction potency. Offspring of male *ubi*:*creERT2* to female *ubi*:*Switch* crosses were induced with either *trans*-4-OHT or Endoxifen consistently at 50% epiboly. Imaging was performed at 4 dpf. Double-positive embryos were placed lateral (anterior to the left) and an image was taken of the whole embryo applying the same settings. Fluorescence was quantified using an automated Macro in ImageJ. Scale bar 500 μm. (C) To address a potential tissue bias due to possibly restricted drug penetration and recombination, we performed transverse vibratome sections of 4 dpf *ubi*:*creERT2*;*ubi*:*Switch* embryos induced under the same conditions as the embryos used for epi-fluorescence measurements (scale bar 50 μm). The depicted example images show switching in pectoral fin tissue after 4-OHT induction; merged: EGFP, mCherry and DAPI.

**Fig 2:**
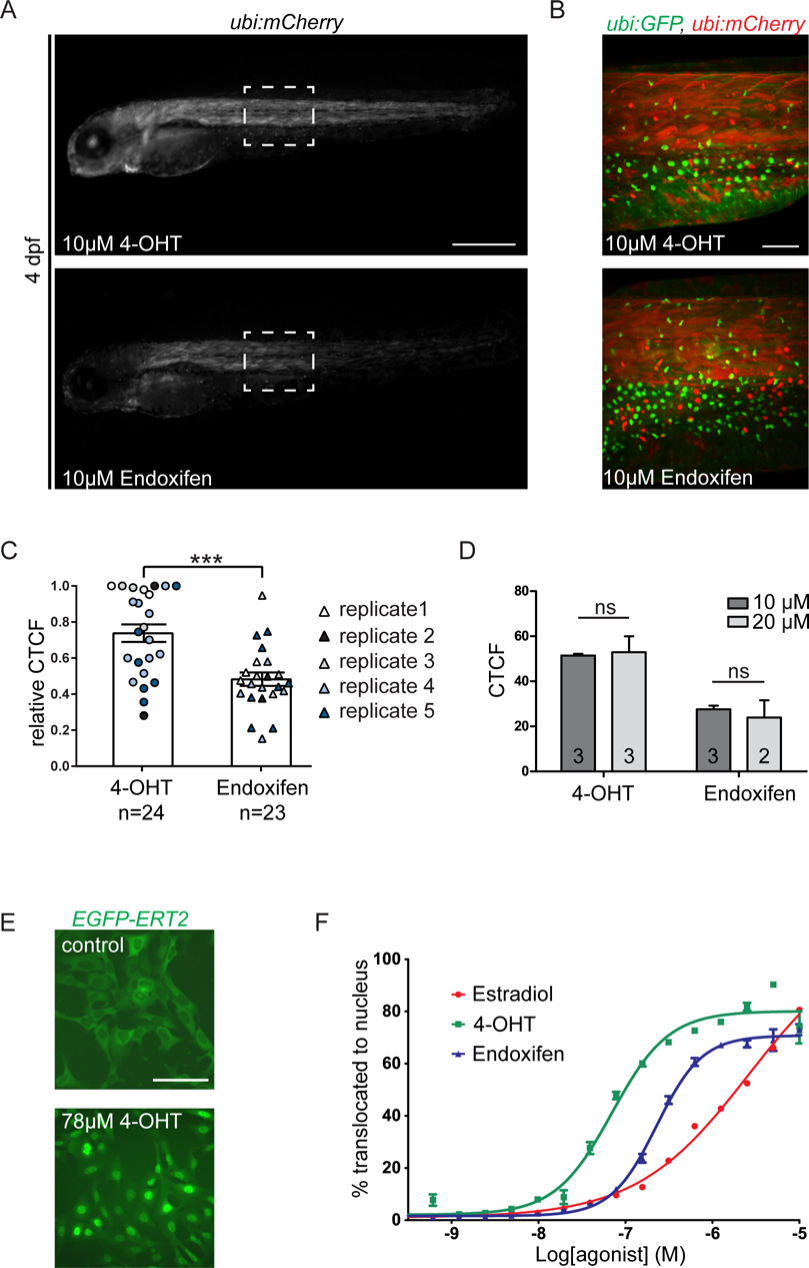
Endoxifen potently induces CreERT2 activity in zebrafish and ERT2 reporters in cell culture. (A) Fluorescence intensity was quantified in *ubi*:*creERT2*;*ubi*:*Switch* embryos induced with freshly dissolved *trans*-4-OHT and Endoxifen (both 10 μM) as a measurement for drug potency (scale bar 500 μm). (A, B) Both compounds efficiently confer CreERT2 mediated recombination as seen in the whole embryo and maximum intensity projections of the trunk imaged with the Zeiss Z.1 lightsheet microscope. (A) scale bar 500 μm, (B) 100 μm. Representative images are shown for each condition. (C) Quantifications of the fluorescence show that Endoxifen induces CreERT2 activity with approximately half the potency (two-tailed, unpaired t-test, p = 0.0001). (D) Switching experiments were done at saturated conditions; increasing the concentration does not increase potency neither for *trans-*4-OHT nor Endoxifen (two-way ANOVA, column p-value 0.8232). (E) *EGFP-ERT2* cells were treated with different concentrations of E2, *trans*-4-OHT, and Endoxifen. Cells were imaged after 20 hours post treatment using High-content imaging system Operetta (Perkin Elmer). Representative images are shown (scale bar 100 μm). (F) Data from multiple fields were analyzed using Columbus software (Perkin Elmer) and the percentage of translocation was plotted using GraphPad Prism software. Endoxifen acts with slightly lower potency than *trans*-4-OHT, but both compounds are more potent than the native ligand E2.

CreERT2 recombination in zebrafish embryos is routinely performed with *trans*-4-OHT at a final concentration of 5–10 μM in E3 embryo medium from native *trans*-4-OHT stocks stored at 10mM in either DMSO or EtOH [19]. To assess CreERT2-based *loxP* recombination potency upon stimulation, we devised a quantitative imaging and analysis workflow (Fig 1A–1C): as genetic basis to read out CreERT2 activity, we combined the established zebrafish transgenics for *ubi*:*creERT2* and *ubi*:*lox-EGFP-lox-mCherry* (*ubi*:*Switch*) [13], in which the respective transgenes are ubiquitously expressed (Fig 1A); double-transgenic embryos maintain mCherry fluorescence in all descendant cells that had active CreERT2 upon induction. Addition of freshly dissolved *trans*-4-OHT (from 10 mM stock in DMSO) at a final concentration of 10 μM in E3 medium to double-transgenic embryos at 50% epiboly (approximately 5.25 hours post-fertilization (hpf)) consistently revealed ubiquitous mCherry expression when whole embryos where imaged at 4 dpf to allow for full *ubi*:*Switch* expression (Fig 1B). We quantified the mCherry fluorescence using Fiji (Fig 1B, see Methods for details). We additionally analyzed possible tissue bias of recombination in transverse vibratome sections of fixed embryos (Fig 1C). Using this workflow, we found that akin to 10 μM *trans*-4-OHT, 10 μM Endoxifen potently induced CreERT2 activity (Figs 1A, 2A and 2B). Of note, the perceived and measured induction potency based on mCherry as proxy was consistently half for Endoxifen compared to fresh *trans*-4-OHT (Fig 2C; two-tailed, unpaired t-test, p = 0.0001). Consistent with previous reports [13], 10 μM *trans*-4-OHT added during gastrulation saturated the system, as 20 μM *trans*-4-OHT triggered virtually identical fluorescence values (Fig 2D, two-way ANOVA, column p-value 0.8232).

To test the general applicability of Endoxifen for experimental ERT2 control, we analyzed the potential of *trans*-4-OHT and Endoxifen to mediate nuclear translocation of an EGFP-ERT2 fusion protein in cultured human cells (see Methods). Similarly to the native ligand E2, addition of *trans*-4-OHT potently triggered nuclear translocation of EGFP-ERT2 after 20 hours of treatment (Fig 2E and 2F). Administration of Endoxifen also resulted in efficient nuclear translocation without notable toxicity, but consistently at lower potency compared to *trans*-4-OHT (Fig 2F). This data shows that the Tam metabolite Endoxifen is a less potent, but a functional alternative to *trans*-4-OHT for experimental control of ERT2 fusion proteins across different systems.

**Fig 3:**
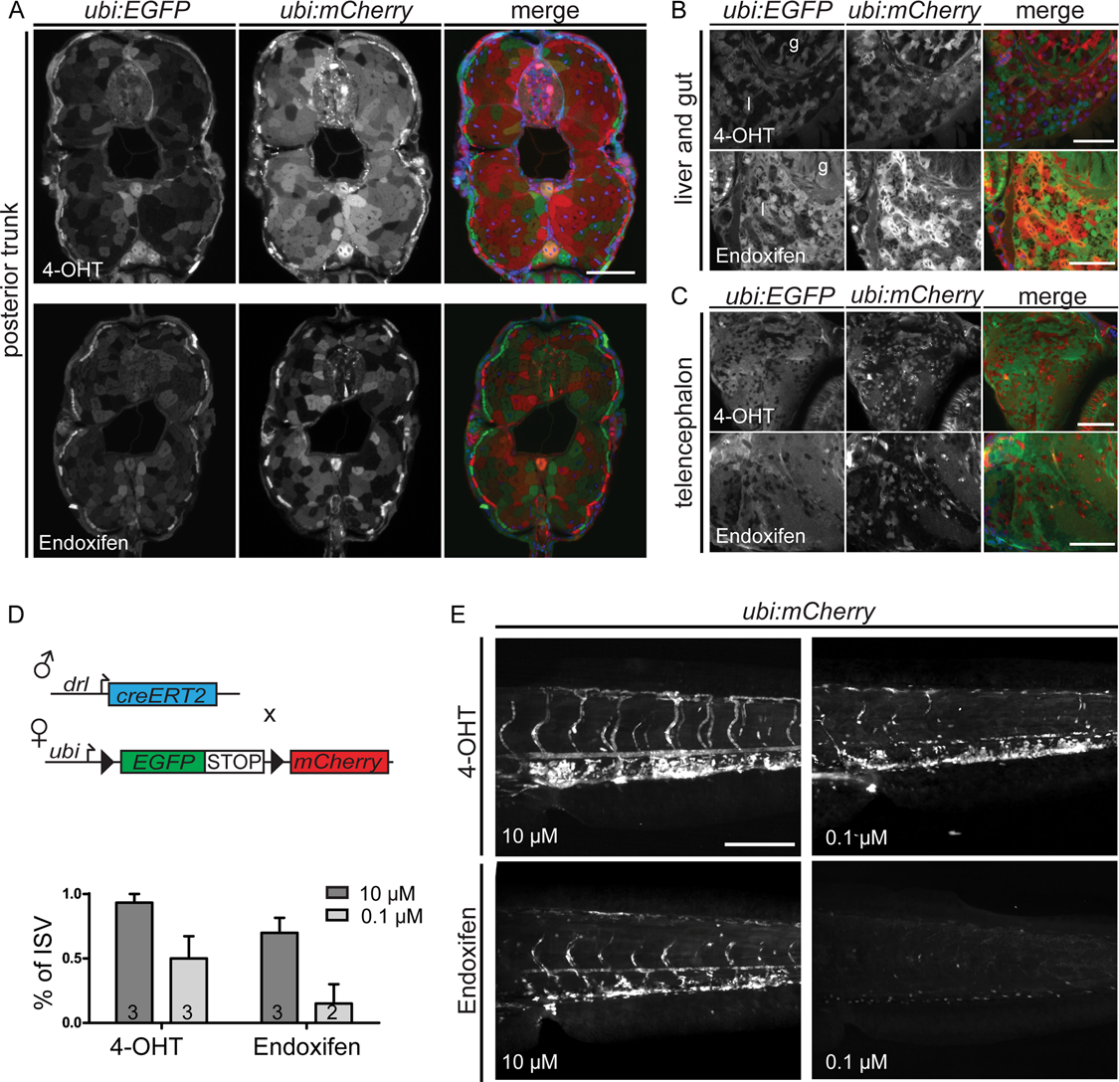
Endoxifen confers CreERT2 mediated recombination at a lower potency in tissue specific lineage tracing experiment. (A-C) Transverse vibratome sections of *ubi*:*creERT2;ubi*:*Switch* at 4 dpf were performed to control for tissue bias in *trans*-4-OHT- versus Endoxifen-induced recombination. (A) Sections of posterior trunk (*trans*-4-OHT n = 13; Endoxifen n = 10), (B) anterior liver (l) and gut (g) (*trans*-4-OHT n = 7; Endoxifen n = 3), and (C) telencephalon (*trans* 4-OHT n = 7; Endoxifen n = 3) show similar switching efficiencies among tissue sections between the two compound treatments. Representative images are shown for each condition. Merged: EGFP, mCherry, and DAPI. (D) To compare *trans*-4-OHT and Endoxifen potency in a tissue-specific lineage tracing experiment, *drl*:*creERT2* transgenics were crossed to *ubi*:*Switch*. (D, E) *trans*-4-OHT induced CreERT2 mediated recombination more potently at saturated concentrations (10 μM) and non-saturated conditions (0.1 μM) compared to Endoxifen. For quantifications shown in D, switched intersomitic vessels (ISV) in *trans*-4-OHT or Endoxifen treated *drl*:*creERT2;ubi*:*Switch* were imaged with the Zeiss lightsheet Z.1 and counted in the maximum intensity projection (scale bars 200 μm). In embryos treated with 10 μM *trans*-4-OHT, all ISVs in the analyzed part of the trunk are mCherry positive with very few parts of individual ISVs unlabeled. Reducing the concentration to 0.1 μM confers sub-optimal switching for clonal analysis. Endoxifen shows lower potency with fewer mCherry positive ISVs. Lowering Endoxifen concentration to 0.1 μM fails to induce efficient CreERT2 activation.

To rule out possible tissue bias of recombination efficiencies by the individual compounds, we performed transverse vibratome sections of 4 dpf embryos induced with either 4-OHT or Endoxifen at 50% epiboly. We assessed whole-body sections (revealing lineages of all germ layers), close-ups of liver and gut endoderm, and of the telencephalon (Fig 3A and 3C). These sections reveal once more the difference in potency of *trans*-4-OHT versus Endoxifen and verify potent recombination in all observed tissues for both compounds.

To complement our findings and to gain a qualitative insight into 4-OHT versus Endoxifen induction efficiency for tissue-specific lineage tracing, we combined *ubi*:*Switch* with the *drl*:*creERT2* driver (Fig 3D), in which the regulatory elements of the zebrafish *draculin* (*drl*) gene specifically drive CreERT2 recombinase in the emerging lateral plate mesoderm and later in cardiovascular lineages [21]. When induced with 10 μM *trans*-4-OHT at shield stage (6 hpf), double-transgenic embryos showed complete mCherry lineage labeling in all intersomitic and main trunk vessels (Fig 3E). Lineage labeling was concentration-dependent, as induction with 0.1 μM *trans*-4-OHT caused mosaic lineage labeling (Fig 3D and 3E). Induction with 10 μM Endoxifen resulted in nearly complete lineage labeling of trunk vessels, with only few intersomitic vessels eluting *loxP* recombination (Fig 3D and 3E). In contrast, induction with 0.1 μM Endoxifen failed to induce efficient switching, confirming the lower potency of Endoxifen (Fig 3D and 3E). Taken together, these observations reveal that both 4-OHT and Endoxifen show similar cell penetrance in early zebrafish embryos and lead to recombination in tissues of all germ layers.

Taken together, our analysis establishes Endoxifen as viable alternative drug to *trans*-4-OHT for ERT2 fusion protein modulation. Endoxifen has a marginally weaker induction capacity than *trans*-4-OHT, which is nonetheless sufficient for basic CreERT2 activity and lineage tracing.

### Endoxifen retains reproducible CreERT2 induction potency even upon prolonged storage

**Fig 4:**
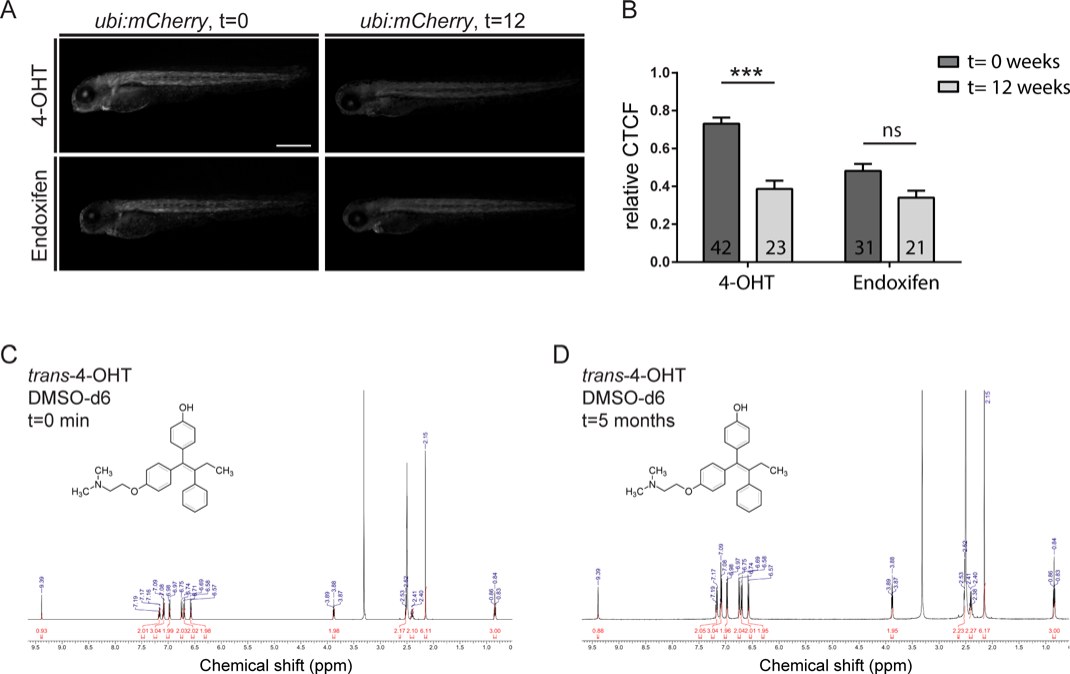
In contrast to Endoxifen, *trans*-4-OHT loses its potency over time independently from isomerization. (A) Switching efficiency was quantified comparing fresh and 12 weeks old *trans*-4-OHT and Endoxifen (scale bar 500 μm). (B) mCherry intensity in embryos treated with 12 weeks old *trans*-4-OHT is approximately half compared to intensities measured in embryos induced with fresh *trans*-4-OHT (two-tailed, unpaired t-test, p<0.0001). The potency of Endoxifen does not change significantly throughout the same time period (two-tailed, unpaired t-test, p = 4598). (C) The ^1^H NMR spectrum of *trans*-4-OHT freshly dissolved in DMSO-d6 reveals the presence of ≥98% of the *trans* isomer. (D) After 5 months storage in DMSO-d6, no appreciable change in isomer composition can be detected by ^1^H NMR.

A key problem of using *trans*-4-OHT for CreERT2 experiments is the propensity of dissolved *trans*-4-OHT stocks to lose induction potency during storage [19]. No alternative compounds have been specifically characterized that would circumvent the problem. We therefore sought to quantify how recombination efficiency changed over time upon prolonged storage of *trans*-4-OHT and Endoxifen in DMSO using standard laboratory conditions (-20°C in the dark). We prepared fresh batches of dissolved *trans*-4-OHT and Endoxifen at 10 mM and distributed the compounds into single-use aliquots for storage to avoid unnecessary compound handling. On day 1 and after 12 weeks, we induced double-transgenic *ubi*:*creERT2*; *ubi*:*Switch* embryos derived from the same parent pool at 50% epiboly with either 10 μM final *trans*-4-OHT or Endoxifen from the original batches. We subsequently quantified mCherry fluorescence at 4 dpf as before (Fig 4A and 4B). This analysis generated a comparison of compound activity of fresh and old *trans*-4-OHT and Endoxifen stocks.

Consistent with previous observations, *trans*-4-OHT kept in 10 mM stocks consistently lost induction potency, and after 12 weeks the residual *trans*-4-OHT activity merely reached half of the starting activity (Fig 4B). These results were reproducible for three independent batches of the compounds and show that storage of dissolved *trans*-4-OHT significantly impairs CreERT2 experimentation (two-tailed, unpaired t-test, p<0.0001). We also observed a drop in induction potency when 4-OHT was stored as 5 mM stocks, suggesting that a range of stock concentrations is affected by this phenomenon (S1 Fig).

Endoxifen stocks maintained high induction potential throughout the test period with a slight, but not significant drop in activity after 12 weeks (two-tailed, unpaired t-test, p = 0.4598) (Fig 4A and 4B). This apparent stability is in stark contrast to the declining activity of *trans*-4-OHT over time (two-way ANOVA, interaction p-value 0.0115).

Altogether, our *in vivo* time course of *trans*-4-OHT and Endoxifen activity for CreERT2 induction provides a first quantification of the commonly observed drop in *trans*-4-OHT activity during storage. Despite its mildly decreased induction capacity, our data also establish Endoxifen as practical alternative for CreERT2 induction in zebrafish and possibly other experimental systems.

### Dissolved *trans*-4-OHT and Endoxifen retain stable isomeric composition.

To resolve the underlying cause for the observable *trans*-4-OHT activity loss, we sought to determine any chemical changes in the stored compounds. *Trans*-4-OHT has been proposed to spontaneously undergo *cis-trans* isomerization in solution and upon light exposure, to be sensitive to hydrolysis, and to be insoluble in water [3,6,18]. Curiously, commercially synthesized Endoxifen starts as an almost equimolar *cis-trans* mixture of isomers, challenging the possibly negative impact of *cis* compounds on CreERT2 activity. An alternative cause for the drop of *trans*-4-OHT activity over time could be a decrease in dissolved compound in the stock solutions.

To investigate concomitantly to the CreERT2 activity assays *in vivo* also the composition of the stock solutions, we performed ^1^H-NMR spectroscopic analysis on age-matched dissolved *trans*-4-OHT and Endoxifen aliquots in DMSO.

^1^H-NMR analysis of the starting compounds established ≥98% *trans*-4-OHT and 45:55 *cis-trans* Endoxifen mixtures of isomers (Fig 4C and S2 Fig). Throughout a period of 5 months, the isomeric composition and structural integrity of dissolved, stored *trans*-4-OHT and Endoxifen remained stable at all analyzed time points (Fig 4D and S3 Fig). To rule out an effect of the solvent, we performed ^1^H-NMR analysis of *trans*-4-OHT dissolved and stored in Ethanol for a period of 4 months as described for DMSO. Similar to DMSO, no difference in the isomeric composition of *trans*-4-OHT could be observed when dissolved in Ethanol (S4 and S5 Figs). While we cannot rule out long-term effects beyond our observation period, our data rule out a storage-caused isomeric change or significant hydrolysis of stored *trans*-4-OHT.

Previous reports noted that 4-OHT becomes unstable upon exposure to light with a high sensitivity to UV light [3,18]. To further corroborate these observations and their impact on regular laboratory handling of Tamoxifen derivatives, we exposed *trans-*4-OHT and Endoxifen stocks to either i) 4 h of daylight at 25°C, ii) 30 min UV light of 254 nm, or iii) 2.5 h UV light of 254 nm. Our NMR analysis revealed that daylight exposure had no impact on *trans*-4-OHT and Endoxifen isomerization or stability (S6 and S9 Figs). In contrast, UV exposure at a wavelength of 254 nm lead to decomposition of both compounds as shown by ^1^H-NMR after 30 min (S7 and S10 Figs) and more pronounced after 2.5 h (S8 and S11 Figs). Our observations confirm that both *trans*-4-OHT and Endoxifen are unstable upon strong UV exposure but also indicate that no special precautions have to be taken when handling the compounds under regular laboratory light conditions.

### *trans*-4-OHT likely precipitates upon storage and is reconstituted upon heating

**Fig 5:**
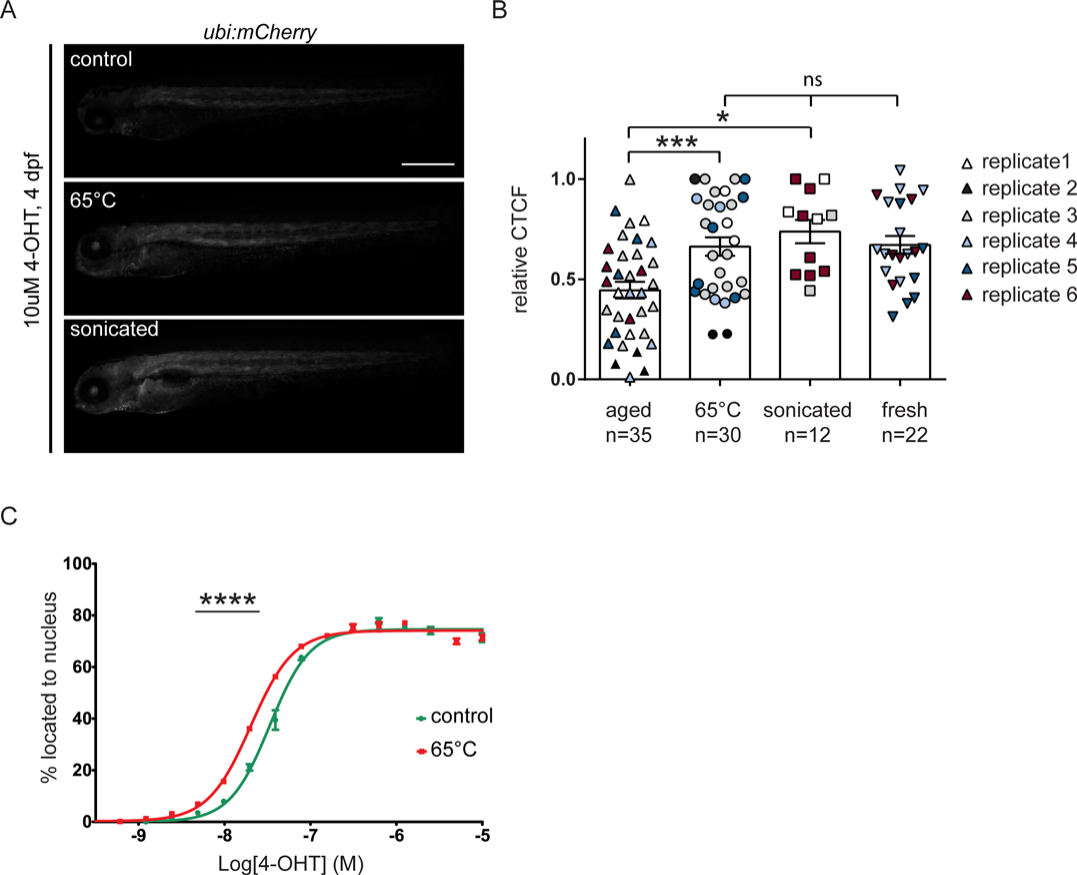
Heating or sonication restores lost *trans*-4-OHT potency. (A) mCherry intensity was quantified in embryos induced with >12 weeks old *trans*-4-OHT that was heated at 65°C or sonicated for 10 min prior to addition to E3 (scale bar 500 μm). (B) Heating or sonicating restores aged *trans*-4-OHT activity compared to both non-heated/non-sonicated compound to levels of the fresh compound (two-tailed, unpaired t-test, aged vs heated p = 0.0008, aged vs sonicated p = 0.0319, heated vs fresh p = 0.9029, sonicated vs fresh p = 0.1684). (C) EGFP-ERT2 cells were treated with different concentrations of control or heated (10 min at 65°C) aged *trans*-4-OHT. Images were taken 20 h post treatment and analyzed using Columbus software. Percentage of translocation was plotted using GraphPad Prism software. Heating moderately improves *trans*-4-OHT potency to induce translocation of EGFP-ERT2 to the nucleus (two-way ANOVA, p<0.0001).

Considering the inert structure of dissolved *trans*-4-OHT, we hypothesized that *trans*-4-OHT potentially could go out of solution over time, and that re-solubilization of *trans*-4-OHT in aged aliquots could restore working stock concentration and *in vivo* CreERT2 induction potential. We next tested the *in vivo* activity of aged *trans*-4-OHT aliquots that we either i) heat-treated for 10 minutes at 65°C, ii) sonicated, or iii) left untreated. Consistent with precipitation, both heat treatment and sonication significantly and repeatedly restored the activity of aged *trans*-4-OHT samples in our zebrafish CreERT2 activity assay (two-tailed, unpaired t-test, aged vs heated p = 0.0008, aged vs sonicated p = 0.0319) (Fig 5A and 5B). We observed no significant differences in recombination efficiency between heated/sonicated aged and fresh *trans*-4-OHT (two-tailed, unpaired t-test, heated vs fresh p = 0.9029, sonicated vs fresh p = 0.1684), suggesting that re-solubilizing aged stocks can restore the compound potency to its original level (Fig 5B). We also detected partial reconstitution upon heating of two year-old 5 mM trans-4-OHT (S1 Fig). Similarly, in the EGFP-ERT2 cell line heat treatment of the aged *trans*-4-OHT at 65°C for 10 minutes lead to a moderate, but significant improvement in the induction of translocation compared to unheated *trans*-4-OHT of the same age (Fig 5C). ^1^H-NMR analysis of aged *trans*-4-OHT that was heated at 65°C revealed no significant changes from the original compound composition (S12 and S13 Figs). Of note, sonicating aged *trans*-4-OHT in glass vials also maintained original compound composition, yet sonication in plastic tubes caused a significant change in compound composition as detected by ^1^H-NMR. While this procedure restores *trans*-4-OHT activity (Fig 5B), sonication in plastic tubes results in a less defined compound composition with possible contaminants and degradation products and should thus be avoided.

Altogether, our findings suggest that dissolved *trans*-4-OHT precipitates out of solution during storage. Definitive confirmation by quantitative HPLC analysis of the samples could not be obtained due to the compound sensitivity to UV exposure in the detector (A. D., C. N., data not shown). Such aged *trans*-4-OHT stocks can be significantly reconstituted by heat treatment. Endoxifen does not suffer major storage issues and remains useable without further treatment.

## Discussion

Recent advances in model organism transgenesis and genome editing have led to a growing interest in genetic recombination experiments. Our results provide two solutions towards performing reproducible CreERT2 experiments in zebrafish and likely other systems with ERT2-based applications.

First, our findings suggest that *trans*-4-OHT precipitates upon prolonged storage in common solvents as shown here for DMSO (similar results were obtained for EtOH, data not shown). Simple heating restores activity, likely by re-solubilizing precipitated *trans*-4-OHT. While sonication does restore activity of aged *trans*-4-OHT, NMR analysis reveals significant and so-far undefined changes in compound composition when performed in plastic tubes; we therefore suggest heat reactivation of aged *trans*-4-OHT stocks. Heating of old stocks circumvents the need to freshly prepare dissolved *trans*-4-OHT for critical recombination experiments, and to ensure high activity of stored *trans*-4-OHT stock solutions upon prolonged storage, potentially saving on reagent costs and labor over time.

Second, we provide *in vivo* evidence for the use of Endoxifen, a Tam and *trans*-4-OHT metabolite, to experimentally control ERT2 fusion proteins. Endoxifen is potent in the same concentration range as *trans*-4-OHT and commercially available at a comparable price, yet remains stable in solution over time, potentially providing a key advantage over the use of *trans*-4-OHT for routine ERT2 experiments. We documented a decreased potency to induce ERT2-based activities that is approximately half the potency of *trans*-4-OHT both *in vivo* CreERT2-mediated recombination (Fig 2A–2D) and cell-based assays (Fig 2E and 2F). The reason for this decreased activity remains unknown. A potential explanation is the reported lower affinity of ERT2 to Endoxifen that might cause the marginally lower activity in our assays, which is nonetheless potent for experimentation. We did, however, not observe increased induction efficiency upon increasing Endoxifen concentration (Fig 2D), as would be expected for a non-saturated switching efficiency due to lower affinity. We interpret this observation as a possible consequence of inhibitory effects of *cis*-Endoxifen which is present in the mixture at a percentage of 45% (S1 Fig) [23]. A pure *trans*-Endoxifen compound could potentially abolish the differences in potency. However, the synthesis is tedious as a ca. 1:1 mixture of *trans* and *cis* isomers of Endoxifen is obtained by following the reported procedures, and thus HPLC separation or equilibration in acidic media is additionally needed to obtain *trans*-Endoxifen in an isomerically pure form [23].

10 μM treatment of *trans*-4OHT and Endoxifen at shield stage reaches all cell layers, which is reflected by recombination of the observed tissue without any obvious bias in the transverse vibratome sections of *ubi*:*creERT2*; *ubi*:*Switch* (Fig 3). Of note, later embryo or adult zebrafish treatment with *trans*-4OHT or Endoxifen, when the animal has formed more complex structures and possible tissue barriers, could cause a possible tissue bias due to different tissue accessibility of the drug.

In practical terms, our observations suggest that *trans-*4-OHT remains the compound of choice for sensitive assays, potentially weak CreERT2 driver transgenes, and when highest levels of *lox* recombination are desired. The use of freshly dissolved or heat treated stored batches is advisable to maximize activity. The significant potency of Endoxifen proposes its use for characterized, well-working CreERT2 drivers and to titrate recombination efficiency when lower mosaicism is required. The key benefit of Endoxifen is its stability in solution, providing a reproducible basis for repeated applications or high-throughput screening.

## Supporting Information

S1 Fig Relative CTCF of embryos treated with *trans*-4-OHT stored at 5 mM. (**A**) Relative CTCF of *ubi*:*creERT2; ubi*:*Switch* embryos treated with fresh *trans*-4-OHT compared to treatment with aged *trans*-4-OHT stored in 5 mM Ethanol, either unheated or heated to 65°C. To compare the activity between fresh and aged *trans*-4-OHT, we used a one-way ANOVA (two-tailed, unpaired t-test, aged vs heated p = 0.0126). (**B-E**) Lateral confocal images of mCherry expression in the anterior trunk of larvae untreated (**B**) or treated with fresh *trans*-4-OHT (**C**), aged *trans*-4-OHT dissolved in 5 mM Ethanol and stored for two years (**D**) and aged *trans*-4-OHT dissolved in 5 mM Ethanol and stored for two years and then heated at 65°C.
(JPG)

S2 Fig ^1^H-NMR analysis of freshly dissolved Endoxifen *(cis/trans* mixture*)* in DMSO-d_6_. (JPG)

S3 Fig ^1^H-NMR analysis of 5 months old Endoxifen *(cis/trans* mixture*)* in DMSO-d_6_. (JPG)

S4 Fig ^1^H-NMR analysis of freshly dissolved *trans*-4-OHT in EtOH-d_6_. (JPG)

S5 Fig ^1^H-NMR analysis of 4 months old *trans*-4-OHT dissolved in EtOH-d_6_. (JPG)

S6 Fig ^1^H-NMR analysis of *trans*-4-OHT exposed to daylight for 4 h at 25°C. (JPG)

S7 Fig ^1^H-NMR analysis of *trans*-4-OHT exposed to UV light for 30 min. (JPG)

S8 Fig ^1^H-NMR analysis of *trans*-4-OHT exposed to UV light for 2.5 h. (JPG)

S9 Fig ^1^H-NMR analysis of Endoxifen exposed to daylight for 4 h at 25°C. (JPG)

S10 Fig ^1^H-NMR analysis of Endoxifen exposed to UV light for 30 min. (JPG)

S11 Fig ^1^H-NMR analysis of Endoxifen exposed to UV light for 2.5 h. (JPG)

S12 Fig ^1^H-NMR analysis of aged *trans*-4-OHT heated at 65°C for 10 min. (JPG)

S13 Fig ^1^H-NMR analysis of aged *trans*-4-OHT sonicated in glass for 15 min. (JPG)

S14 Fig ^1^H-NMR analysis of aged *trans*-4-OHT sonicated in plastic tubes for 15 min. (JPG)
