# Supplementary figures and images for: In Vivo Performance and Properties of Tamoxifen Metabolites for CreERT2 Control
Source: PLoS One. 2016 Apr 14;11(4):e0152989. doi: 10.1371/journal.pone.0152989 (PMC4831813; doi:10.1371/journal.pone.0152989)

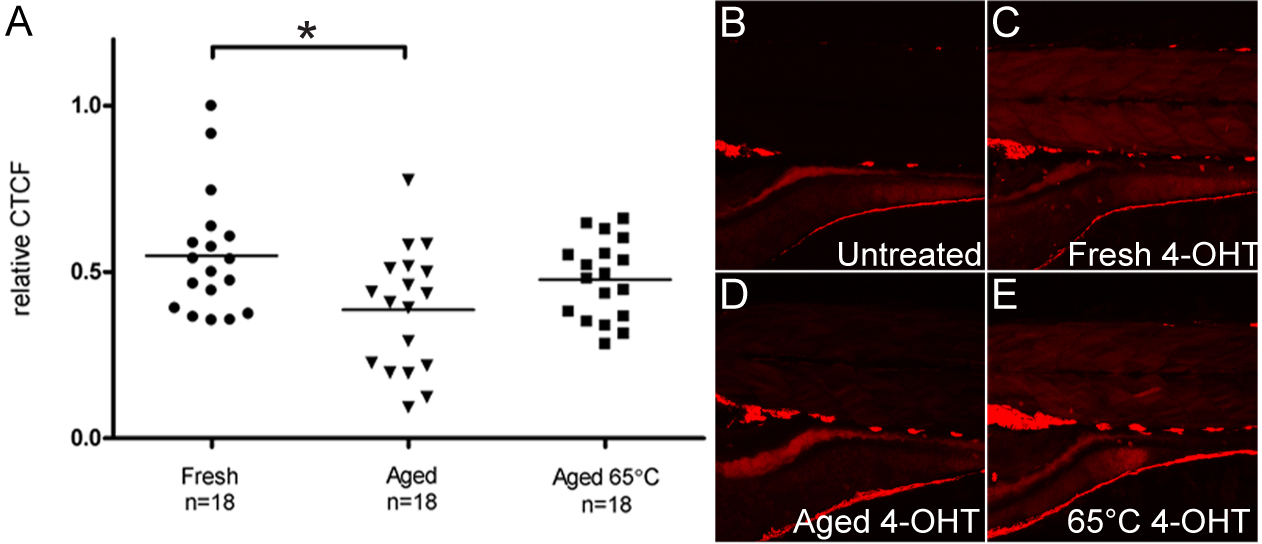

Supplement: S1 Fig — (A) Relative CTCF of ubi:creERT2; ubi:Switch embryos treated with fresh trans-4-OHT compared to treatment with aged trans-4-OHT stored in 5 mM Ethanol, either unheated or heated to 65°C. To compare the activity between fresh and aged trans-4-OHT, we used a one-way ANOVA (two-tailed, unpaired t-test, aged vs heated p = 0.0126). (B-E) Lateral confocal images of mCherry expression in the anterior trunk of larvae untreated (B) or treated with fresh trans-4-OHT (C), aged trans-4-OHT dissolved in 5 mM Ethanol and stored for two years (D) and aged trans-4-OHT dissolved in 5 mM Ethanol and stored for two years and then heated at 65°C. (JPG) [file pone.0152989.s001.jpg]

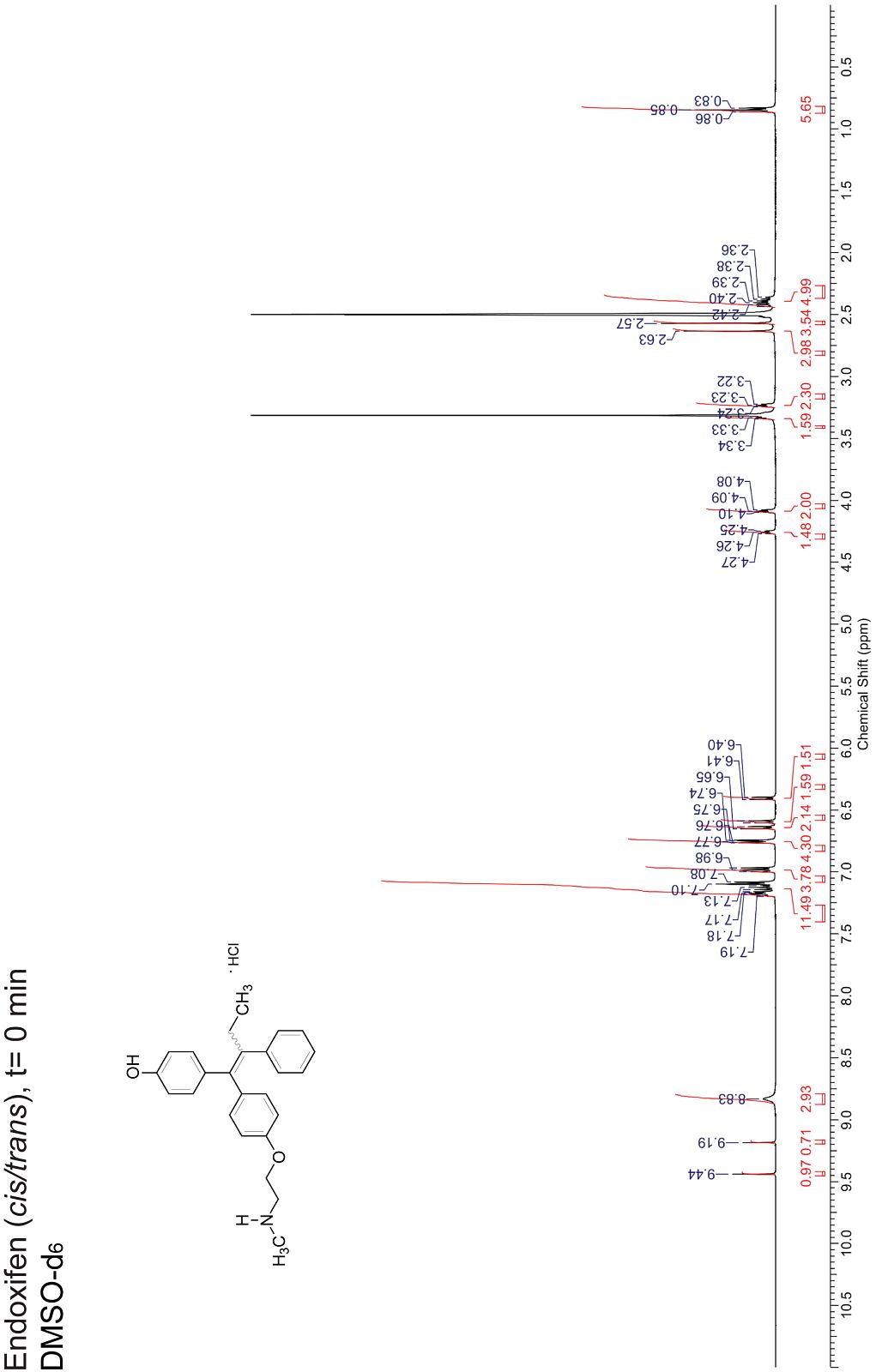

Supplement: S2 Fig — (JPG) [file pone.0152989.s002.jpg]

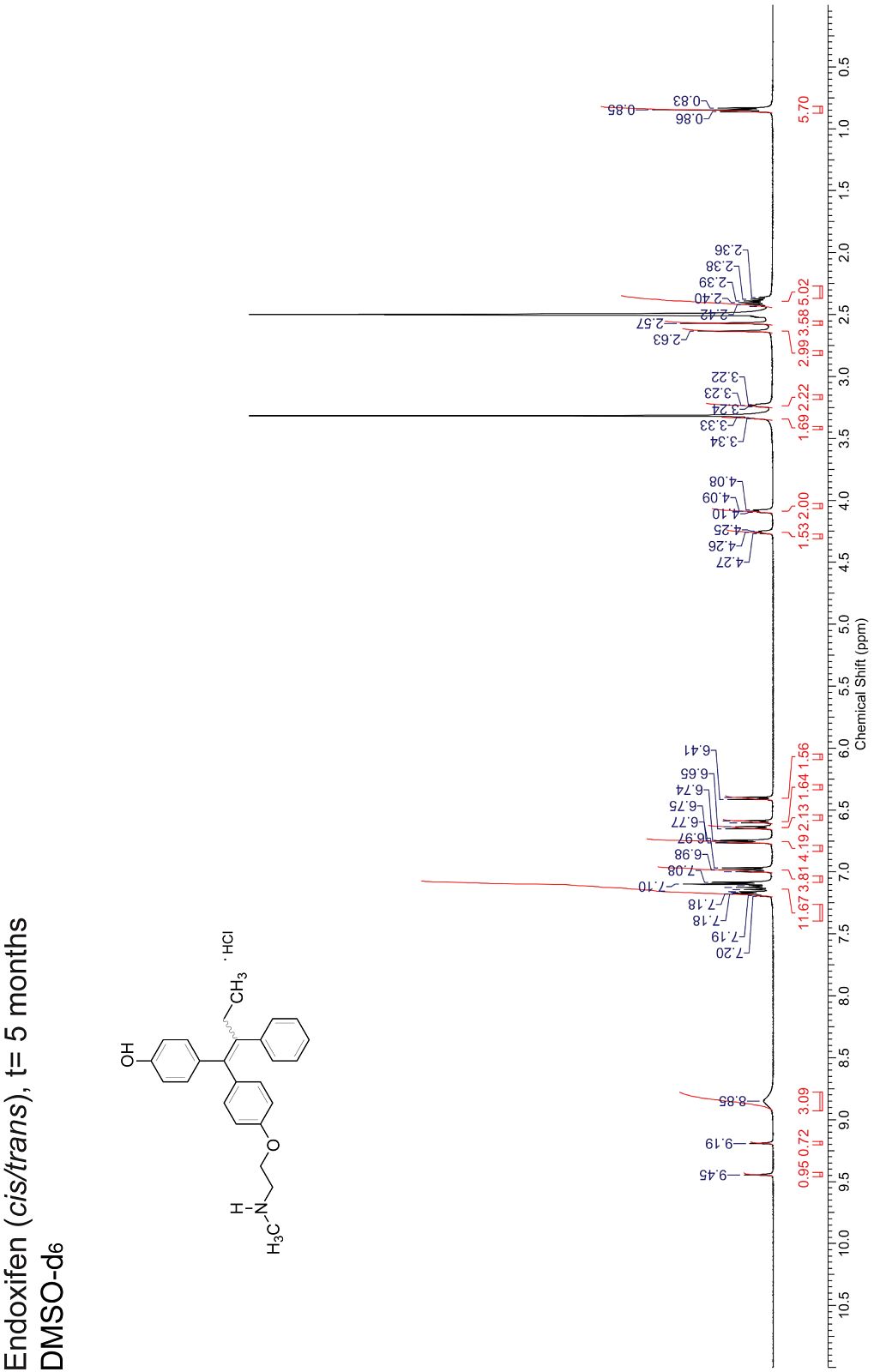

Supplement: S3 Fig — (JPG) [file pone.0152989.s003.jpg]

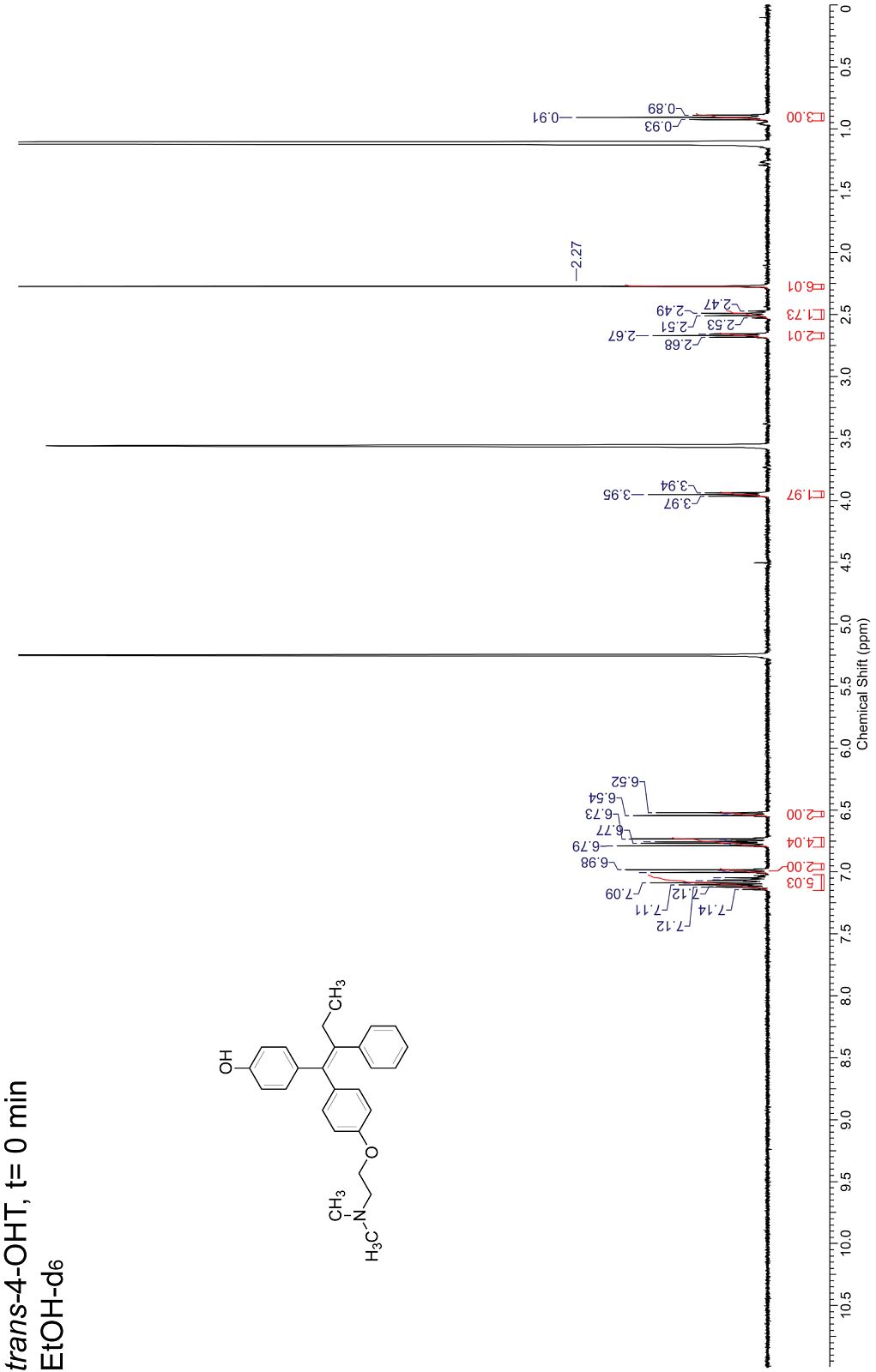

Supplement: S4 Fig — (JPG) [file pone.0152989.s004.jpg]

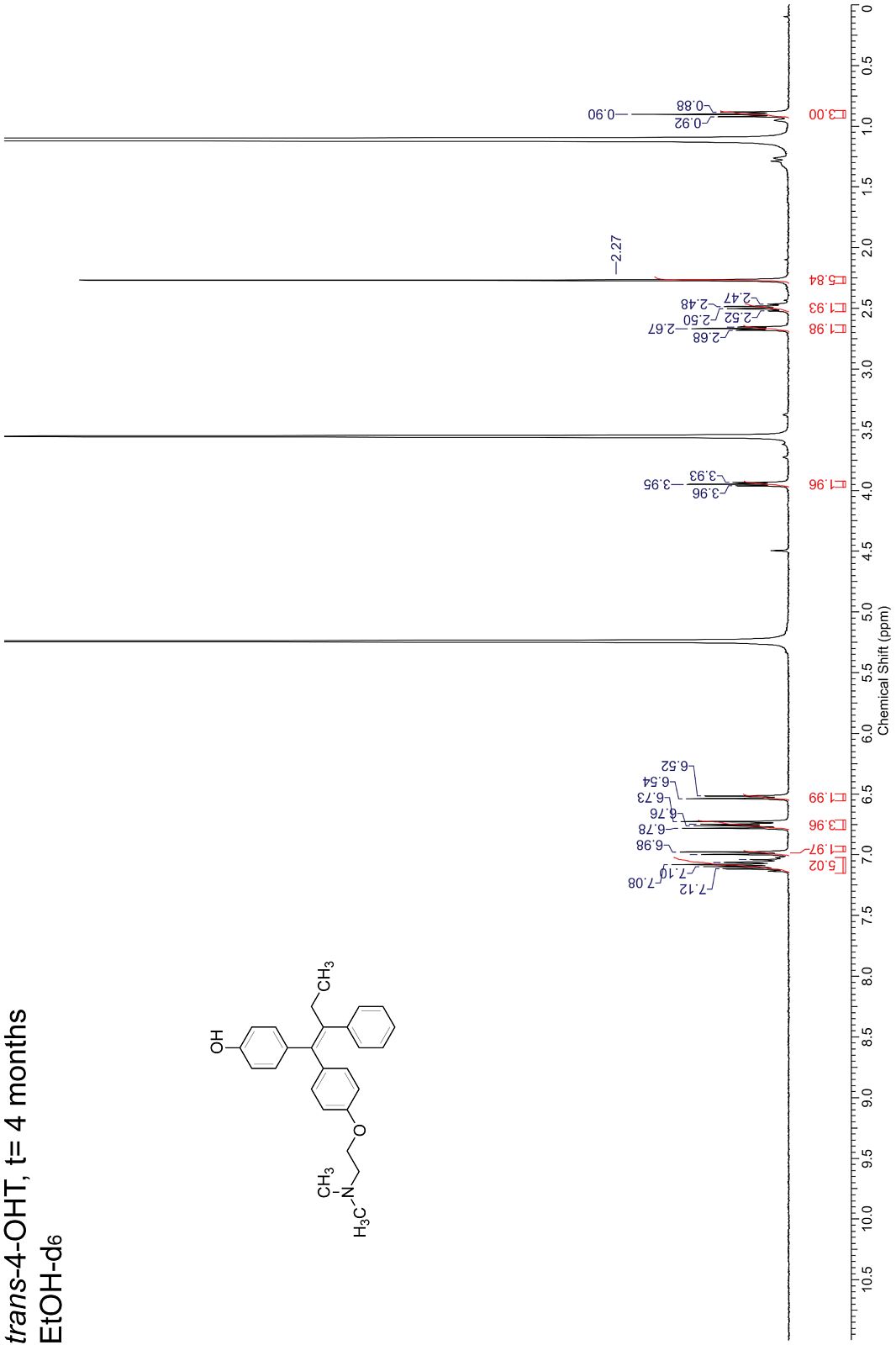

Supplement: S5 Fig — (JPG) [file pone.0152989.s005.jpg]

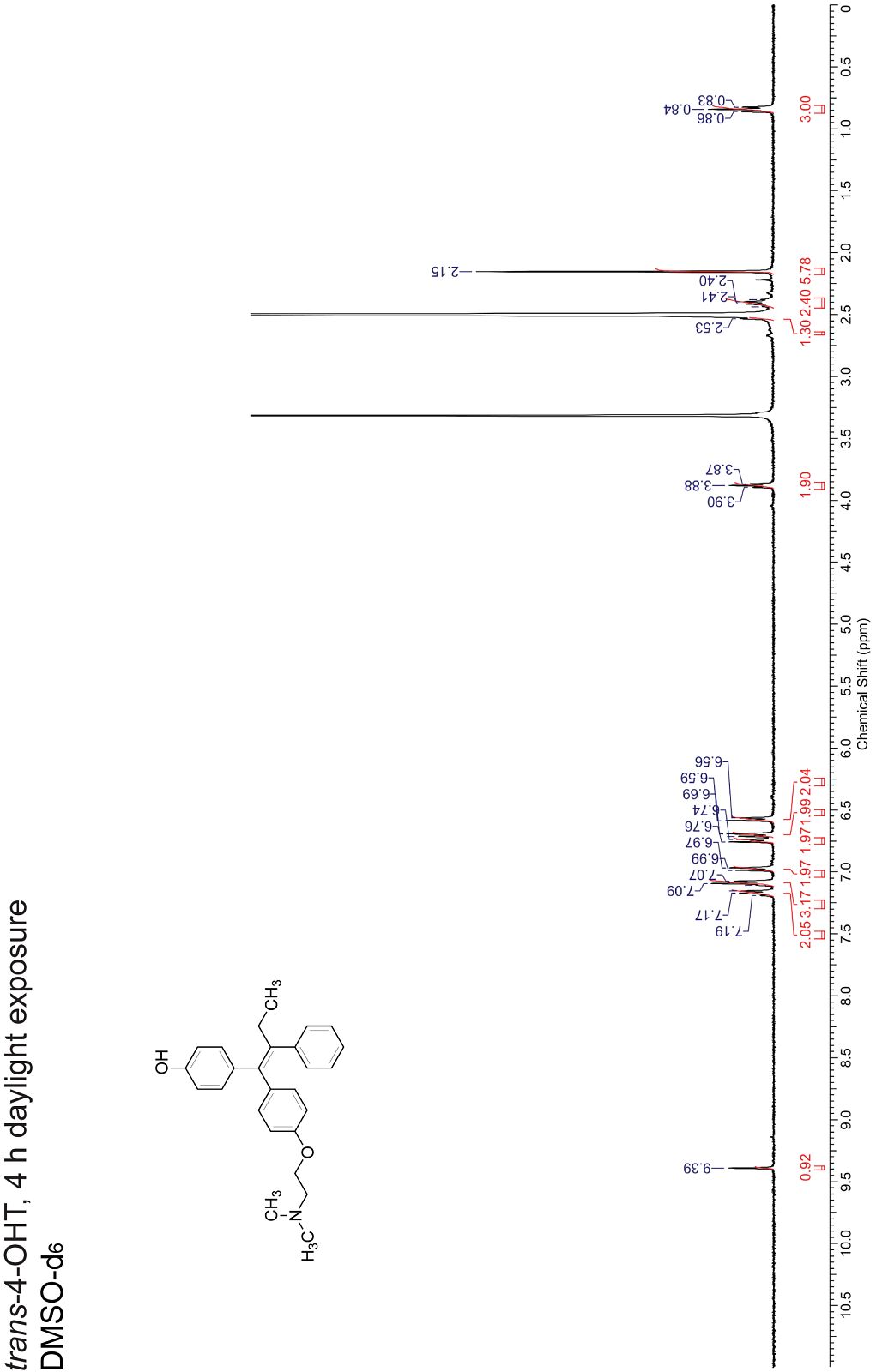

Supplement: S6 Fig — (JPG) [file pone.0152989.s006.jpg]

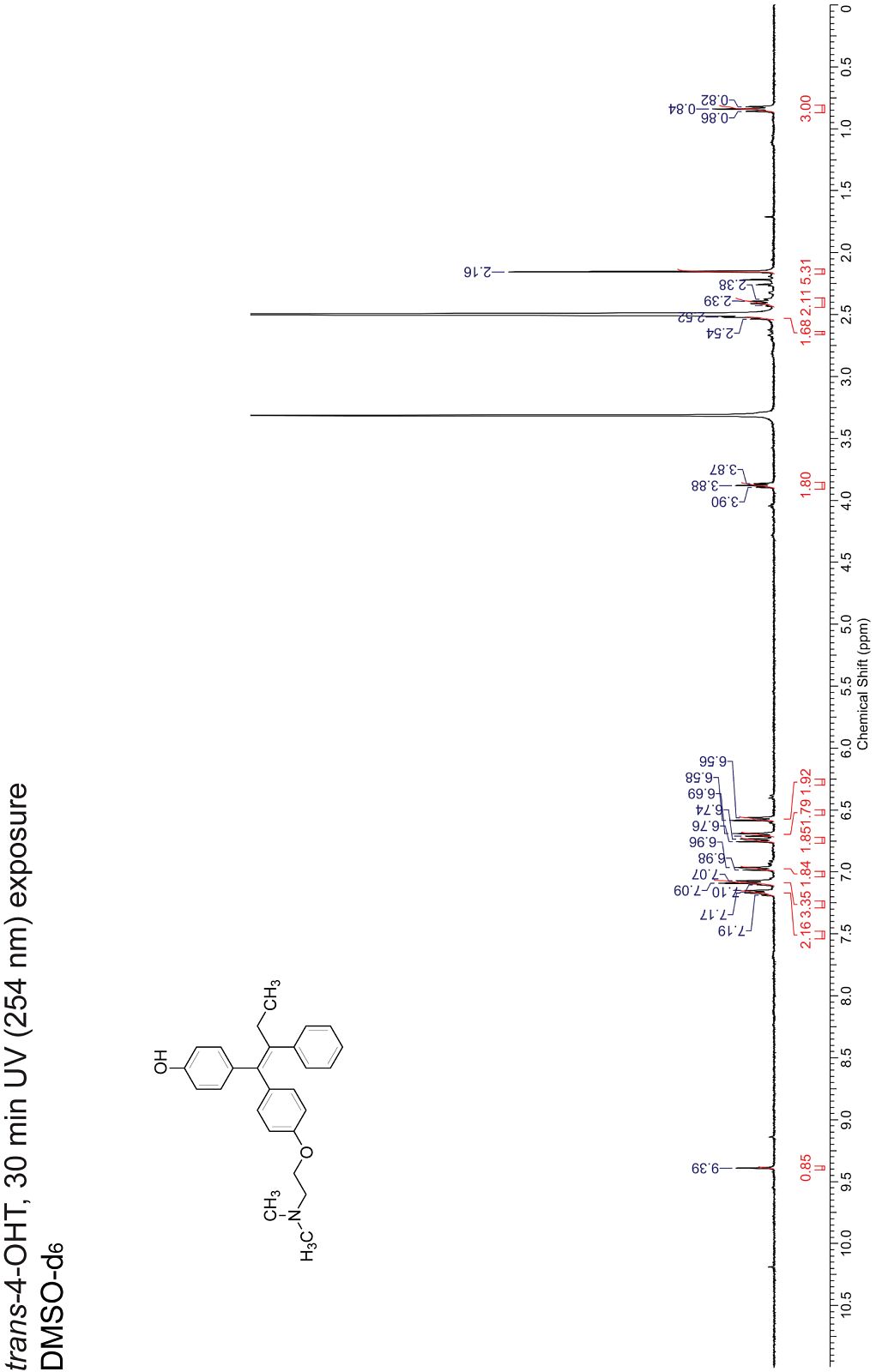

Supplement: S7 Fig — (JPG) [file pone.0152989.s007.jpg]

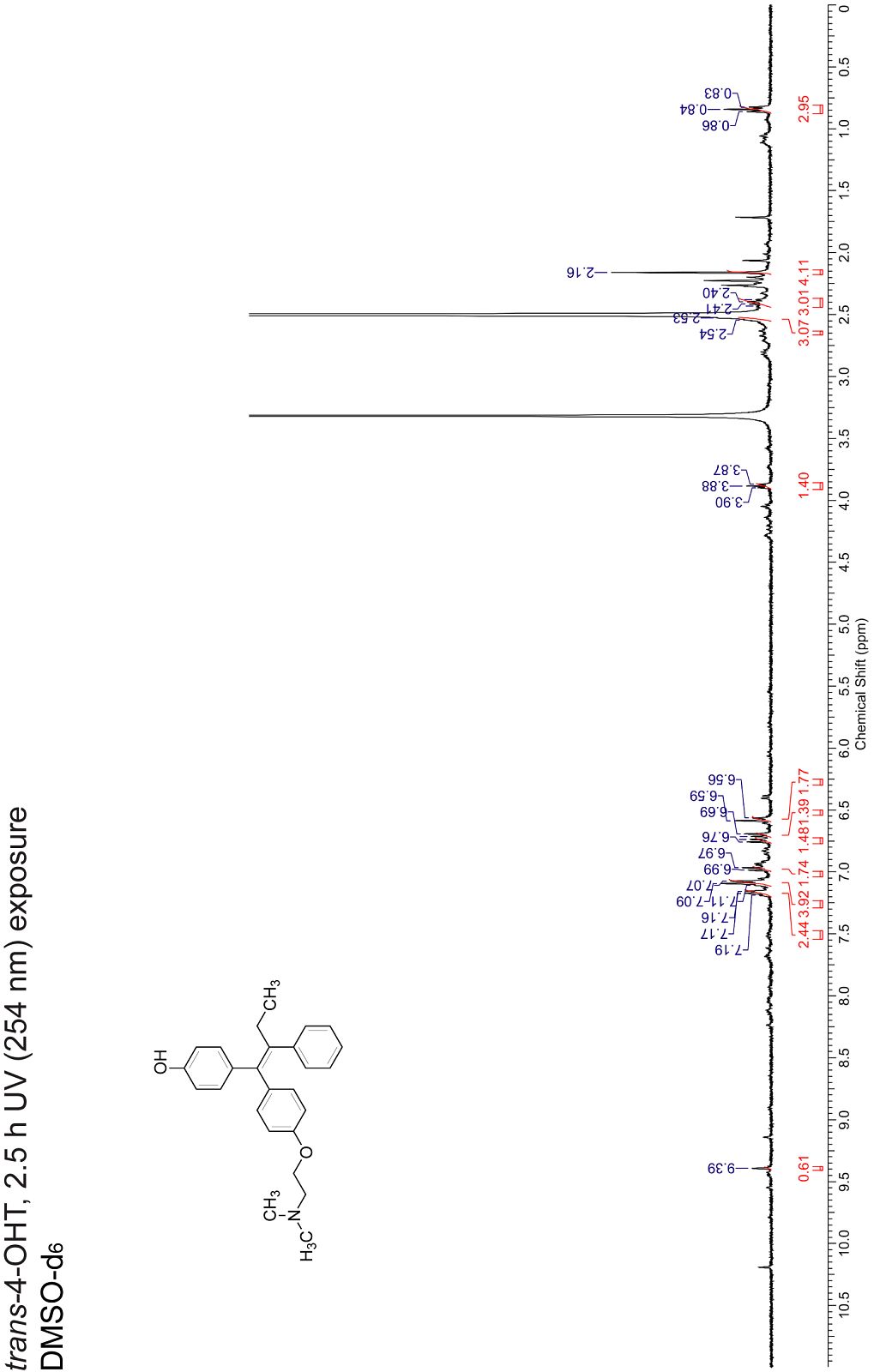

Supplement: S8 Fig — (JPG) [file pone.0152989.s008.jpg]

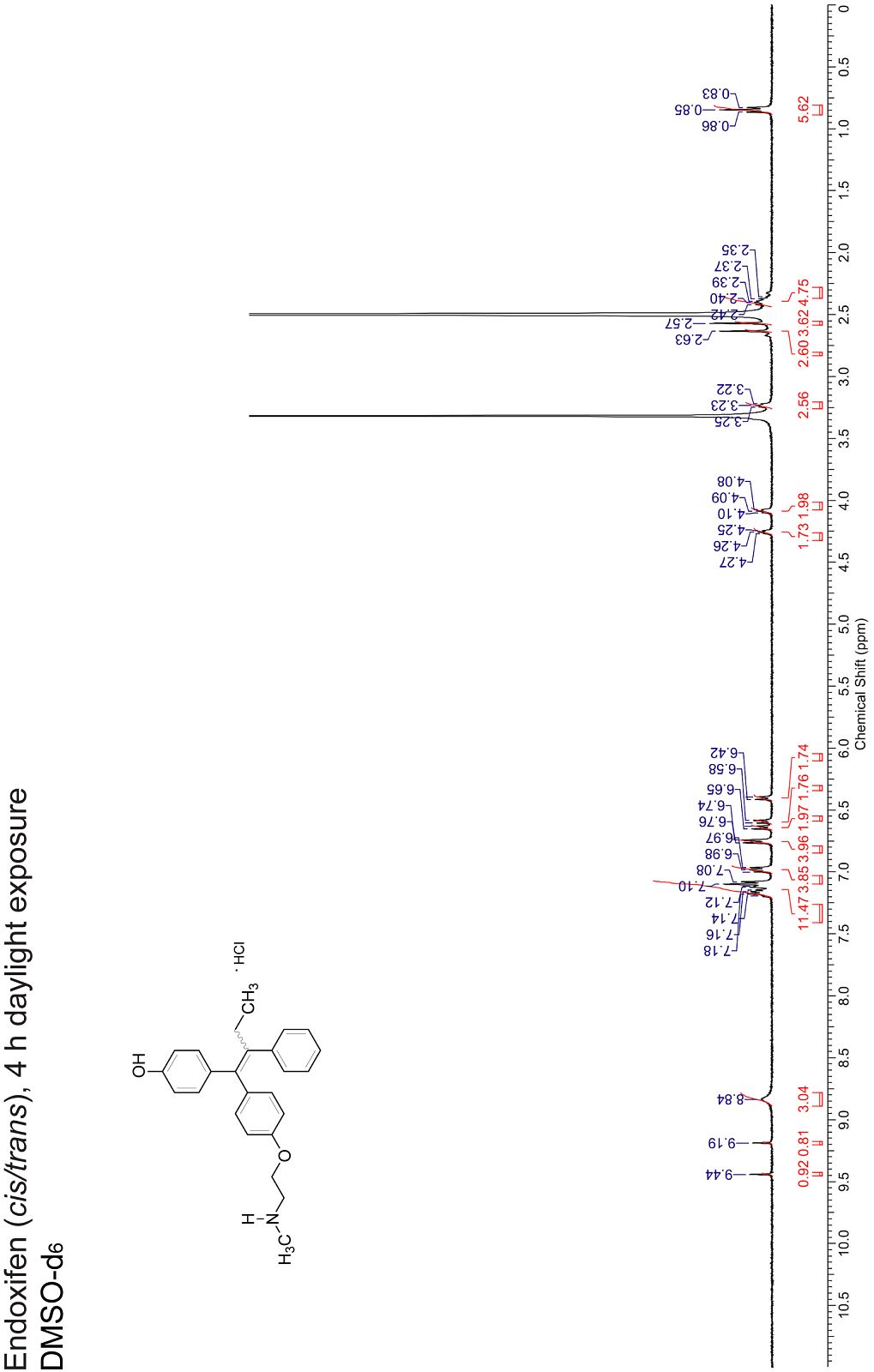

Supplement: S9 Fig — (JPG) [file pone.0152989.s009.jpg]

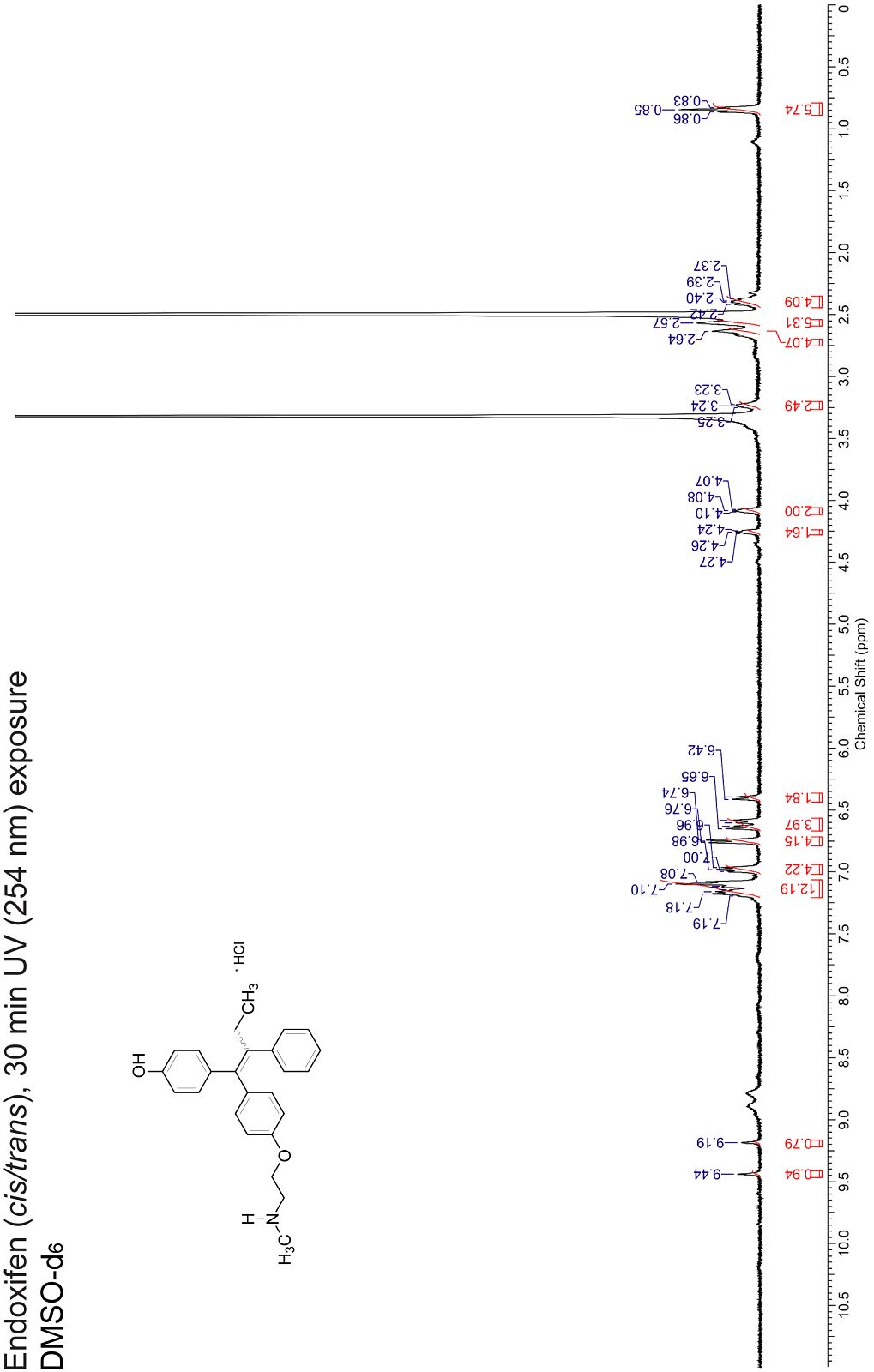

Supplement: S10 Fig — (JPG) [file pone.0152989.s010.jpg]

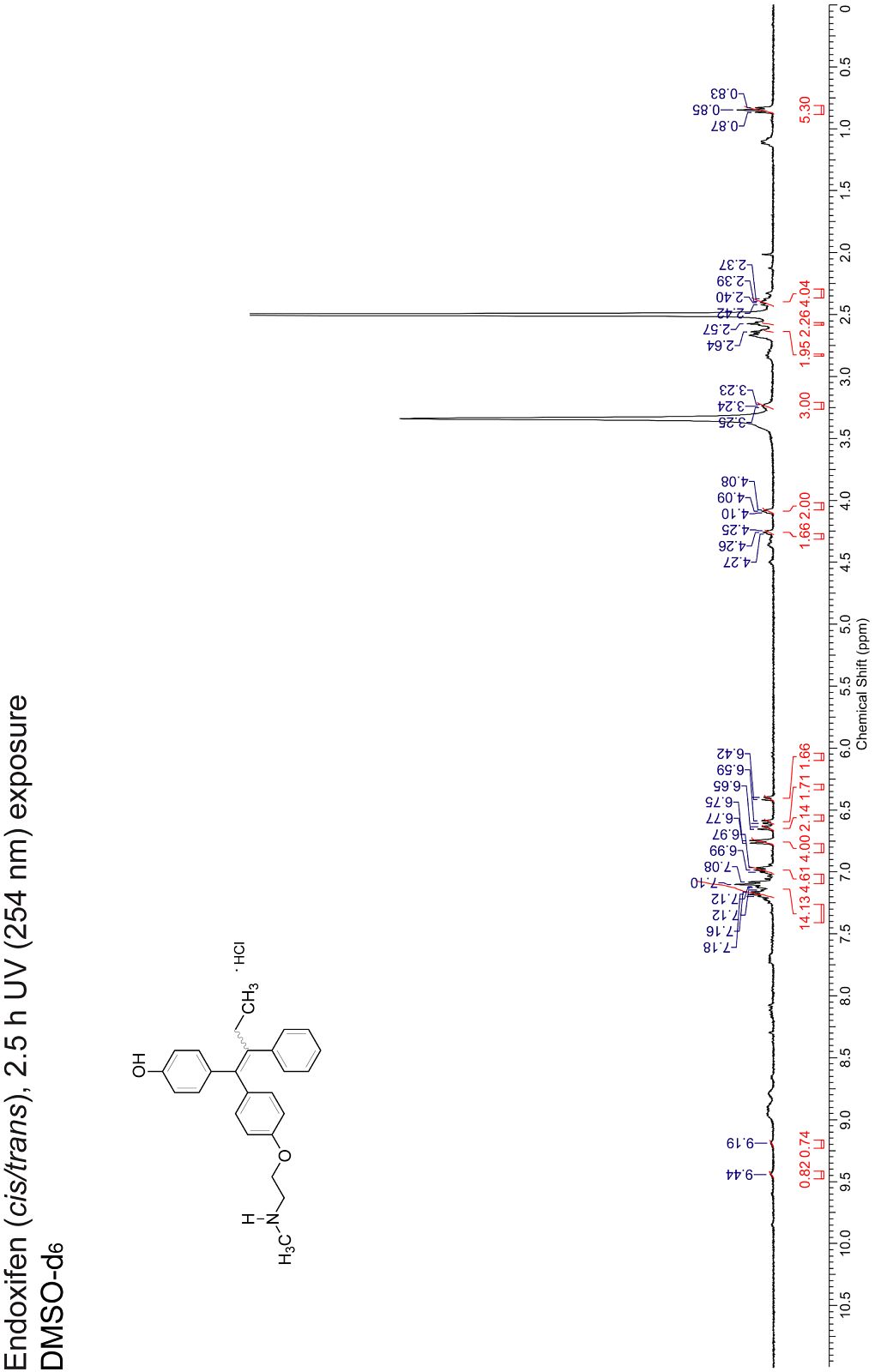

Supplement: S11 Fig — (JPG) [file pone.0152989.s011.jpg]

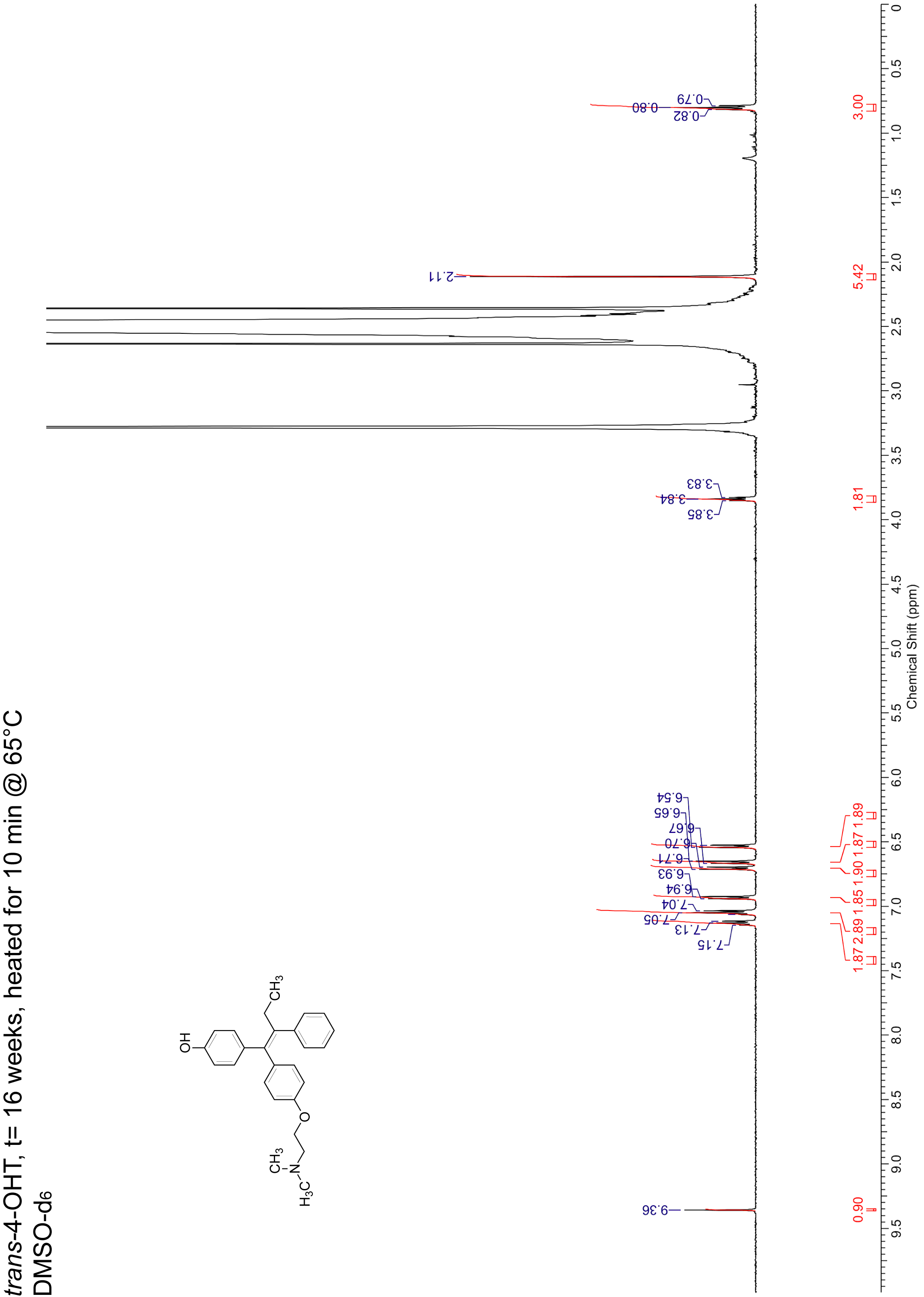

Supplement: S12 Fig — (JPG) [file pone.0152989.s012.jpg]

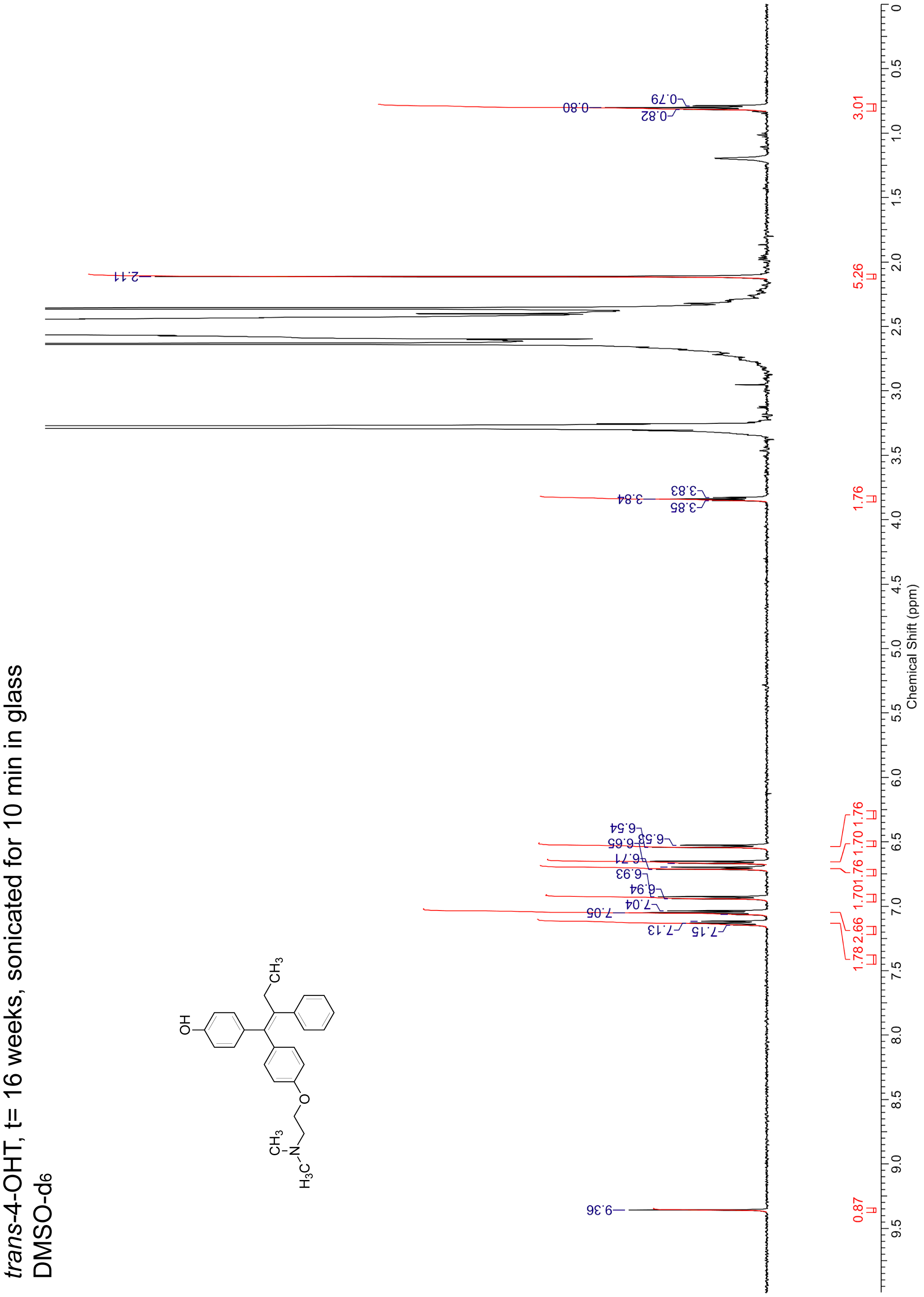

Supplement: S13 Fig — (JPG) [file pone.0152989.s013.jpg]

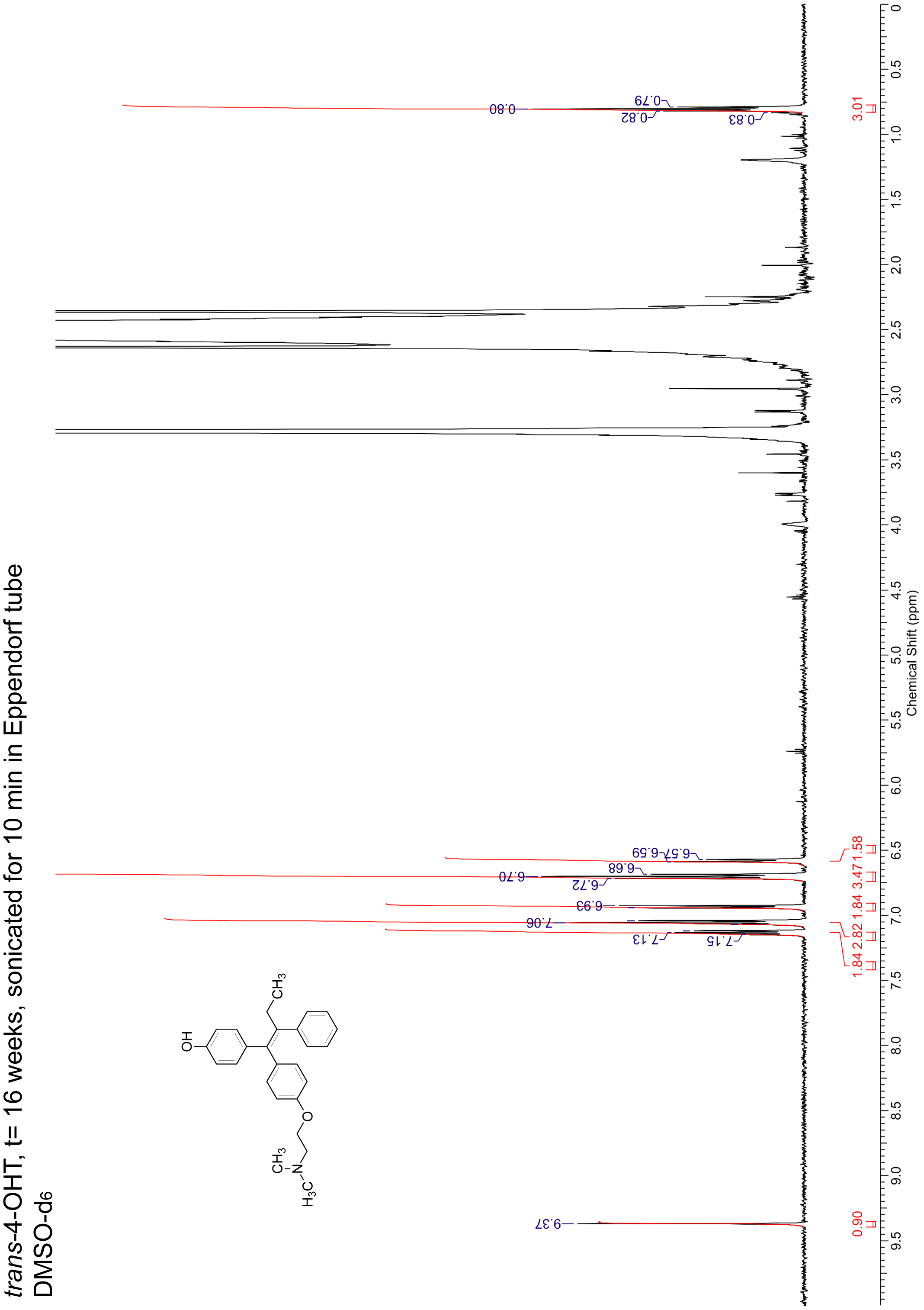

Supplement: S14 Fig — (JPG) [file pone.0152989.s014.jpg]
